# A Curvilinear-Path Umbrella Sampling Approach to Characterizing the Interactions Between Rapamycin and Three FKBP12 Variants

**DOI:** 10.3389/fmolb.2022.879000

**Published:** 2022-07-08

**Authors:** Dhananjay C. Joshi, Charlie Gosse, Shu-Yu Huang, Jung-Hsin Lin

**Affiliations:** ^1^ Research Center for Applied Sciences, Academia Sinica, Taipei, Taiwan; ^2^ Institut de Biologie de l’Ecole Normale Supérieure, ENS, CNRS, INSERM, PSL Research University, Paris, France; ^3^ Institute of Biomedical Sciences, Academia Sinica, Taipei, Taiwan; ^4^ Biomedical Translation Research Center, National Biotechnology Research Park, Academia Sinica, Taipei, Taiwan; ^5^ School of Pharmacy, College of Medicine, National Taiwan University, Taipei, Taiwan; ^6^ College of Engineering Sciences, Chang Gung University, Taoyuan, Taiwan

**Keywords:** rapamycin, FKBP12, umbrella sampling simulations, molecular dynamics, free energy calculation, hydrogen bond

## Abstract

Rapamycin is an immunosuppressant macrolide that exhibits anti-proliferative properties through inhibiting the mTOR kinase. In fact, the drug first associates with the FKBP12 enzyme before interacting with the FRB domain of its target. Despite the availability of structural and thermodynamic information on the interaction of FKBP12 with rapamycin, the energetic and mechanistic understanding of this process is still incomplete. We recently reported a multiple-walker umbrella sampling simulation approach to characterizing the protein–protein interaction energetics along curvilinear paths. In the present paper, we extend our investigations to a protein-small molecule duo, the FKBP12•rapamycin complex. We estimate the binding free energies of rapamycin with wild-type FKBP12 and two mutants in which a hydrogen bond has been removed, D37V and Y82F. Furthermore, the underlying mechanistic details are analyzed. The calculated standard free energies of binding agree well with the experimental data, and the roles of the hydrogen bonds are shown to be quite different for each of these two mutated residues. On one hand, removing the carboxylate group of D37 strongly destabilizes the association; on the other hand, the hydroxyl group of Y82 is nearly unnecessary for the stability of the complex because some nonconventional, cryptic, indirect interaction mechanisms seem to be at work.

## Introduction

Protein–ligand interactions are central in modern drug-discovery, and their characterization by various approaches is crucial for better drug development. In this regard, computational investigation is one of the ways to acquire a deeper understanding of the interactions of interest. In particular, molecular dynamics (MD) simulations provide the physical connection between the structure and the function of biomolecules (Karplus and McCammon, 2002), especially at the atomic level; therefore, MD simulation-based techniques can often cast insights into such interactions, especially in the early stage drug-discovery (Mobley and Gilson, 2017). In addition to conformational dynamics of the interacting molecules, MD simulations are also employed to estimate thermodynamic properties such as the relative binding free energy and the binding affinity. Major ongoing challenges in this field are related to the reliability, the accuracy, and the rapidity of the estimation method/approach.

A reliable estimation of the free energy difference between thermodynamically well-defined end states of interacting molecules is one of the major goals of computational biophysics. Umbrella sampling along a chosen reaction coordinate, followed by potential of mean force calculations, is one of the ways widely used to estimate binding affinities (Torrie and Valleau, 1977; Kästner, 2011). However, umbrella sampling along predefined vectorial reaction coordinates followed by potential of mean force (PMF) profile constructions has some serious concerns in performing reliable energetic calculations (Doudou et al., 2009). Recently, we discussed them for the protein–protein systems and proposed a naïve multiple-walker approach, in which independent umbrella sampling simulations were conducted without predefined vectorial reaction coordinates. We observed similar large variations in the values of converged PMF profiles, resulting from different curvilinear paths. The variations were attributed as due to the different excessive dissipations in different paths taken, and therefore the lower-bound PMF was chosen, and by introducing a correction term derived from statistical mechanics, the standard free energy of binding was estimated for the protein–protein complex system (Joshi and Lin, 2019). The estimations were in good agreement with the experimental values for the barnase–barstar complex. Furthermore, the revealed mechanistic details from our simulations, e.g., the physical pathways/trajectories of dissociation/association, were quite consistent with two major physical pathways that were determined from several milliseconds-long adaptive molecular dynamics simulations reported by Plattner et al. (2017). Thus, the proposed approach is quite useful in maintaining a suitable balance between estimation of binding energetics and revealing underlying mechanistic details of the dissociation reaction within much less computational cost than the brute force approaches. Since the sampling is enhanced along a spontaneously evolved (i.e., non-predefined) curvilinear physical trajectories, the overall approach is referred to as curvilinear-path umbrella sampling (CPUS) approach. In the present paper, we extend our previous work to the interactions of a drug with its protein partner.

This implementation of CPUS will be validated on FKBP12, a model used in computational ligand binding studies for more than 20 years (Pearlman and Connelly, 1995; Lin et al., 2002; Lin et al., 2003; Sun et al., 2003; Swanson et al., 2004; Fujitani et al., 2005; Lee and Olson, 2006; Olivieri and Gardebien, 2011; Dickson and Lotz, 2016; Nerattini et al., 2016). This enzyme participates in protein folding by catalyzing the isomerization of amide bonds adjacent to proline residues (Hamilton and Steiner, 1998; Galat, 2013). Discovered because it could bind the immunosuppressant macrolide FK506, it was further shown that it could also complex with Rapamycin, a parent drug sharing the same pharmacophore and similar therapeutic indications (Van Duyne et al., 1993; Hamilton and Steiner, 1998; Galat, 2013). It later turned out that the inhibition of the FKBP12 enzymatic activity was irrelevant to account for the pharmaceutical properties of the two macrolides: In fact, this protein only potentializes the binding of each drug to its effective cellular target through the formation of a specific ternary complex, with calcineurin for FK506 and with mTOR for rapamycin (Choi et al., 1996; Banaszynski et al., 2005; Galat, 2013). Apart from its biological significance, the choice of FKBP12 as a model in the 1990s can be explained by its small size (107 residues) and its compact structure, both key factors at a time where computational power was limited. Moreover, several X-ray crystal structures were quickly released, in free form as well as in complex with FK506 and rapamycin (Van Duyne et al., 1991; Van Duyne et al., 1993; Wilson et al., 1995). Additionally, with time, a huge amount of biophysical data have been accumulated from thermodynamic and kinetic measurements on the complexation reaction (Bierer et al., 1990; Holt et al., 1993; Bossard et al., 1994; Connelly et al., 1994; Luengo et al., 1995; DeCenzo et al., 1996a; Schuler et al., 1997; Wagner et al., 1998; Graziani et al., 1999; Dickman et al., 2000; Banaszynski et al., 2005; Wear et al., 2007; Wear and Walkinshaw, 2007; Shor et al., 2008; Kozany et al., 2009; Wu et al., 2011; Tamura et al., 2013; Singh et al., 2015; Lu and Wang, 2017; Kostrz et al., 2019; Wang et al., 2019) to NMR investigations on the protein dynamics (Sapienza et al., 2011; Yang et al., 2015; Solomentsev et al., 2018).

As far as *in silico* studies are concerned, FKBP12 is extensively used as a model system to study protein–ligand interaction energetics. For instance, the relaxed complex scheme (Lin et al., 2002) was employed to estimate interaction energetics of FKBP12 with several different ligands (Lin et al., 2003). Another study using FKBP12 was performed to establish the groundwork for the end-point free energy methods, in which the theoretical framework was proposed to calculate the association free energy (Swanson et al., 2004). A study on estimation of absolute binding free energy calculations of FKBP12 and eight ligands was carried out, where the Bennett acceptance ration (BAR) method was employed in the direct calculations (Fujitani et al., 2005). The FKBP12•ligand system was used to estimate the binding free energies with two ligands, 4-hydroxy-2-butanone and FK506, respectively. Although the necessity of sampling along curvilinear paths was mentioned, the theoretical framework for the free energy estimation was developed only for linear/vectorial paths, and with some quadratic approximations for the variance along the principal axis (Swanson et al., 2004; Lee and Olson, 2006). The CPUS approach can be considered as an alternative approach without such approximations.

**FIGURE 1:**
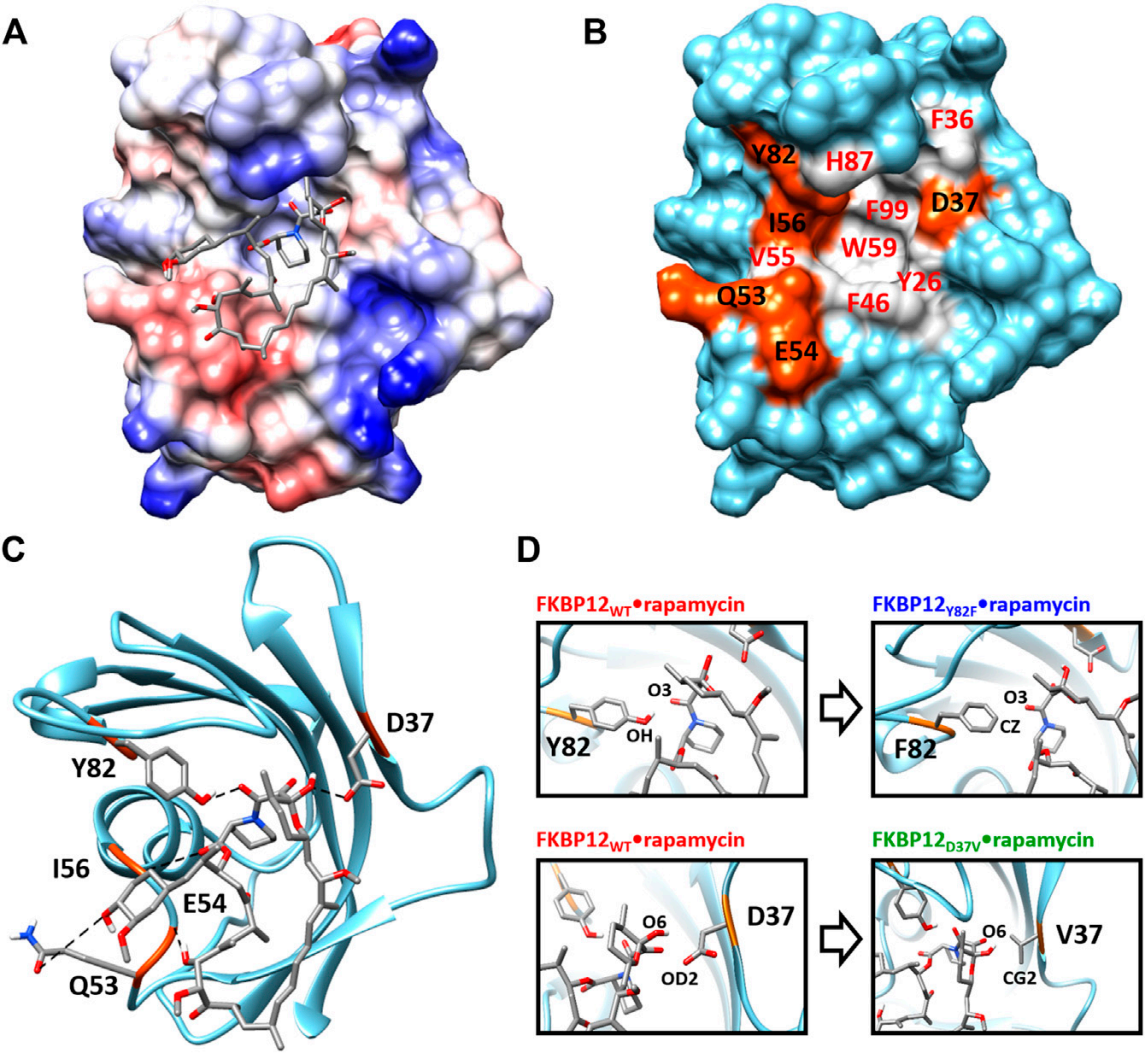
Structure of the FKBP12•rapamycin complex as determined in PDB 1FKB. **(A)** Coulombic surface representation of the protein with the ligand as sticks. **(B)** Surface representation of the protein alone with the hydrogen-bond forming residues in orange and the hydrophobic residues in gray—coloring according to the LIGPLOT diagram provided as Supplementary Figure S1 (Wallace et al., 1995). **(C)** Ribbon representation of the protein with the ligand as sticks. The five hydrogen bonds formed between FKBP12 and rapamycin are shown as dashed lines. **(D)** Close-up view on the Y82 and D37 residues and resulting starting conformation obtained after either the Y82F or the D37V substitution.

From a structural point of view, the macrolide binding site (Van Duyne et al., 1991; Van Duyne et al., 1993) significantly overlaps with the FKBP12 catalytic cleft, in agreement with the observed catalytic inhibition (DeCenzo et al., 1996a; Ikura and Ito, 2007). Rapamycin, the only ligand we will study here, binds in the cavity located between the short α-helix and the five-stranded β-sheet that is wrapped around it. More specifically, the drug pipecolinyl ring is deeply buried inside the protein (Figure 1A) and is involved in hydrophobic interactions with the aromatic side-chains of residues Y26, F46, W59, and F99 (Figure 1B; Supplementary Figure S1)—see (Van Duyne et al., 1993; Sun et al., 2003) for lists of the atom-pair contacts. In addition to auxiliary hydrophobic interactions with some of the amino acids surrounding the cavity (i.e., F36, V55, I56, H87, Y82—Figure 1B), rapamycin is retained at the FKBP12 surface through five hydrogen bonds. Of the hydrogen bond-implied residues (Figures 1B,C), D37, I56, and Y82 are also positioned on the rim, whereas Q53 and E54 are more distant. Since the first three amino acids are the most conserved (Van Duyne et al., 1993; Hamilton and Steiner, 1998; Galat, 2013), they seemed the most attractive for performing an *in silico* mutagenesis program aiming at demonstrating the strengths of the CPUS approach. More precisely, our goal was to test if we could predict the influence of H-bond removal on the stability of the FKBP12•rapamycin complex. We finally selected to target D37 and Y82 because they represent significantly contrasting examples of the contribution of hydrogen bonding to ligand binding. Indeed, experimental measurements have demonstrated that the D37V substitution strongly affects the protein–drug interaction (DeCenzo et al., 1996a; Singh et al., 2015; Kostrz et al., 2019), whereas the Y82F substitution does not (Connelly et al., 1994; Pearlman and Connelly, 1995; Kostrz et al., 2019) (Figure 1D). Incidentally, beyond the determination of the binding free energies for the wild-type protein and the two mutants, we expect that the MD trajectories produced by CPUS will enable us to understand the intriguing role of the H-bond formed between Y82 and rapamycin, a bond that has an atypical geometry (Van Duyne et al., 1993), which displays a clear signature in NMR spectroscopy (Yang et al., 2015), but whose elimination has nearly no impact on the affinity (Bossard et al., 1994; DeCenzo et al., 1996b).

## Materials and Methods

### System Modeling

The FKBP12_WT_•rapamycin binary complex was simulated using the PDB 1FKB crystal structure as a starting conformation (Van Duyne et al., 1991). For the complexes involving the Y82F and D37V mutants, we simply carried out *in silico* mutagenesis using the swapaa module of the Chimera molecular viewing platform (Pettersen et al., 2004) (Figure 1D). Thus, we here clearly assume that none of the two amino acid substitutions results in any significant structural change with respect to the wild-type reference. Such an assertion has already been harnessed in the FKBP12_Y82F_ case, crystallographic evidences being provided to support it (Pearlman and Connelly, 1995)—despite our efforts, we could not retrieve the original data from any of the usual repositories. As far as FKBP12_D37V_ is concerned, we performed ^1^H-^15^N hetero-nuclear single quantum coherence NMR measurements and chemical shift perturbation analysis to back up our starting hypothesis (Supplementary Figure S2) (Williamson, 2018). Omitting the substituted amino acid, only three residues display changes larger than the mean values of the all changes plus two standard deviations: one is facing D37 in the adjacent β-strand and two are in the loop just downstream of the mutation site. As a consequence, we ruled out the possibility of any large-scale rearrangement.

The partial charges on the atoms of rapamycin were derived with the RESP scheme, calculated with Gaussian03 at the level of HF/6-31G basis set. The coordinates from the wild-type crystal structure and from the two mutant models were then processed using the antechamber and LEaP programs of the AMBER software suite (Case et al., 2005; Salomon-Ferrer et al., 2013). The well solvated system was built with 22926 TIP3P waters; 42 K^+^ and 43 Cl^−^ ions were added to neutralize the system and to mimic a 100 mM salt concentration. The box dimensions were 112.4 Å × 81.6 Å × 90.7 Å. The improved ff14SB force field along with the general amber force field (GAFF) (Wang et al., 2004) was used for all bonded and non-bonded parameters (Maier et al., 2015).

### Molecular Dynamics Simulations

Production runs were conducted using the Graphical Processing Unit (GPU RTX 2080Ti) implementation of the AMBER pmemd program. All MD simulations were conducted under NPT conditions with SHAKE-enabled 2-fs time steps. The particle mesh Ewald (PME) algorithm of electrostatics was employed, and the non-bonded interaction cutoff was set to 10.0 Å. Prior to production run, all three systems were subjected to energy minimization, heated to 295 K, and then equilibrated for 100 ps. MD trajectories were analyzed using the cpptraj program of AmberTools-20 (Case et al., 2020) and several in-house shell and python scripts.

### Multiple Walker Curvilinear-Path Umbrella Sampling Simulations

**FIGURE 2:**
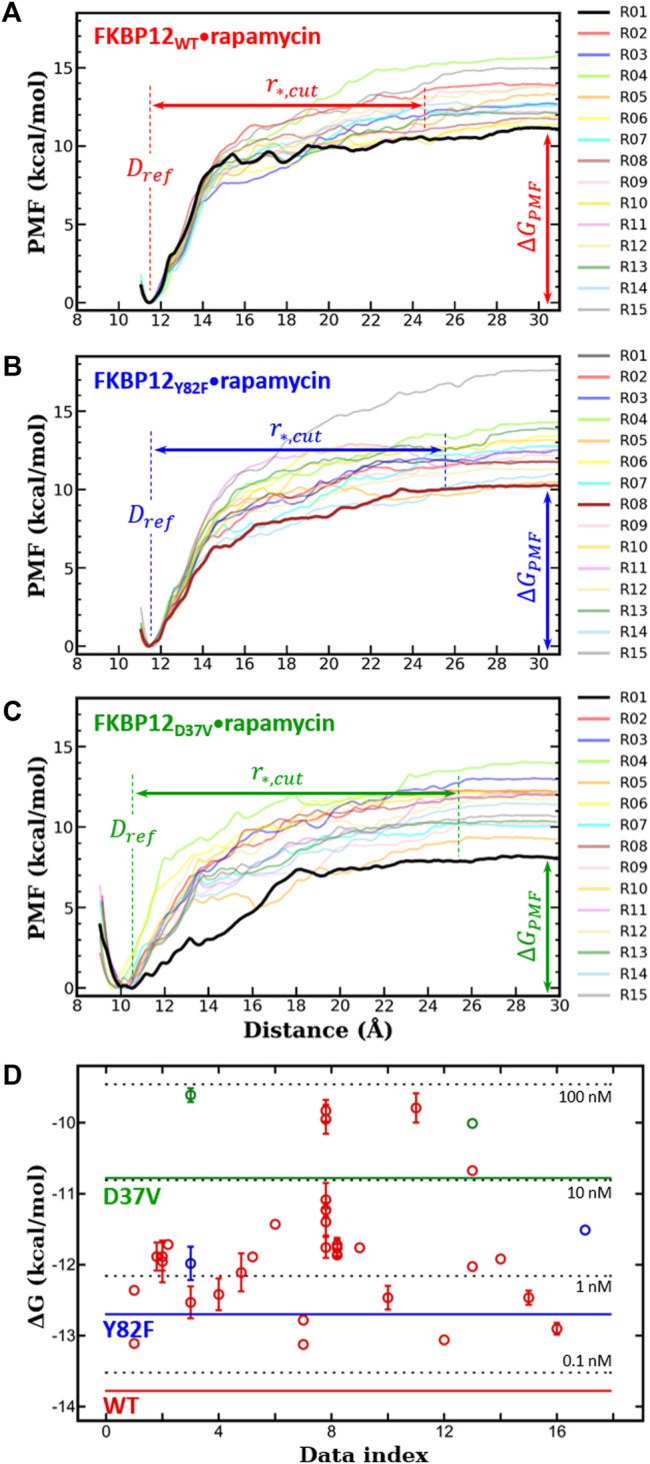
*In silico* determination of the binding free energies for the three variants of FKBP12 + rapamycin ⇄ FKBP12•rapamycin reaction and comparison with experimental data. **(A)** PMF profiles issued from the 15 runs of CPUS MD simulations performed on the FKBP12_WT_•rapamycin complex at $T^{sim}$ = 21.85°C. The lower-bound PMF profile is highlighted in bold, and snapshots taken during this particular dissociation process are displayed in Supplementary Figure S3. Furthermore, we have indicated with double-headed arrows the associated cutoff separation distance, $r_{*,cut}$ , and binding free energy, $\Delta G_{PMF}$ (see Supplementary Table S1 for all 15 numerical values). **(B)** Same plots and views for FKBP12_Y82F_•rapamycin (see Supplementary Figure S4 for the snapshots associated with the lower-bound PMF). **(C)** Same plots and views for FKBP12_D37V_•rapamycin (see Supplementary Figure S5 for the snapshots associated with the lower-bound PMF). **(D)** Collation of the corrected binding free energies obtained with the MD CPUS approach, $\Delta G_{bind}^{0}$ as horizontal lines, and of measurements retrieved from the literature and extrapolated at $T^{sim}$ , $\Delta G_{exp}^{corr}$ as open circles (see Table 1 and Supplementary Table S2 for the corresponding numerical values). Red markers refer to the wild-type FKBP12, blue ones refer to the Y82F mutant, and green ones refer to the D37V mutant. Data have been sorted along the x-axis according to their publication year; moreover, to emphasize on possible biases due to individual practices, we have clustered together all measurements coming from a same laboratory. The four horizontal, black, and dashed lines indicate the ΔG values associated with the 0.1, 1, 10, and 100 nM dissociation equilibrium constants.

The successive steps necessary to implement multiple-walker umbrella sampling simulations along non-predefined curvilinear paths, as well as the corresponding theoretical framework and data treatment procedures, have been described in a previous article (Joshi and Lin, 2019). In brief, before each CPUS run, a short 10 ns-long unbiased MD simulation was conducted and the mean distance between the centers of geometry (CoG) of both FKBP12 and rapamycin was computed so as to provide a reference bound distance, $D_{ref}$ . These CoG are defined by all Cα atoms for FKBP12 and all heavy atoms for rapamycin in the DISANG file, an input to the pmemd. The last frame of this unbiased MD simulation is used as the starting conformations for the CPUS simulation. In the umbrella sampling simulation, the reaction coordinate was chosen as the distance D between the CoGs. The reaction path was divided into N windows (i.e., the umbrella windows), and D was restrained using a biasing potential with a spring constant of k = 10.0 kcal/mol/Å^2^ so as to keep the interacting molecules in the ξ ^th^ distance window at distance D_ξ_. The sampling in each umbrella window was enhanced sequentially so that the physical trajectory of dissociation could evolve spontaneously. To do so, the last conformation from each sampled window was chosen as the starting conformation for the next window for sampling enhancement. The distance sequence took the form ${\left\{D_{\xi }\right\}}_{\xi =1}^{\xi =N}\text{ with }D_{\xi }=\;D_{ref}+\delta \left(\xi -1\right)$ and δ the distance per step, set to 0.1 Å. The PMF profile was constructed using well-established weighted histogram analysis methods (WHAMs) (Kumar et al., 1992; Grossfield, 2013), which removed the contribution of biased potential (Figures 2A–C). Finally, $\Delta G_{PMF}$ was chosen as the PMF value corresponding to the final distance of the constructed profile, $D_{final}$ . Incidentally, the PMF profile was completed for distances smaller than $\;D_{ref}$ by conducting the umbrella sampling in the backward direction for 10 or 20 windows (depending on the cases).

For each of the FKBP12 variants, 15 umbrella sampling simulations were carried out independently by assigning different starting velocities, i.e., setting ig = −1 in the pmemd input file. Sampling in each distance window was enhanced for 1.0 ns, and CPUS MD simulations were 3.15 µs-long in total.

### PMF Correction to Standard Binding Free Energy

To determine the standard free energy of binding, $\;\Delta G_{bind}^{0}$ , each pair composed of a CPUS MD trajectory and of the associated PMF profile was further processed according to Eq. 1 (Joshi and Lin, 2019),

$$\Delta G_{bind}^{0}=\;\Delta G_{PMF}-k_{B}T\ln\left[\left(\frac{4\pi r_{*,\;cut}^{2}}{V_{0}}\right)\int_{bound}dr\;e^{-\beta A_{r}}\right]$$ (1)

with $r=D-D_{ref}$ being the separation distance, $V_{0}$ being the standard state volume (1 663 Å^3^), $A_{r}$ being the PMF value at the separation distance r , and $\beta =1/k_{\text{B}}T$ . The evaluation of the second term on the right-hand side requires to know $r_{*,cut}$ , i.e., the separation distance at which the interaction between the protein and its ligand vanished. To determine this cutoff, an interface interaction analysis was conducted using the linear interaction energy (LIE) module of the AMBER cpptraj program (Roe and Cheatham, 2013). The first cancellation of the van der Waals component provided $r_{*,cut}$ . Then, using home-grown python and UNIX shell scripts, the bound integral included in the second term of Eq. 1 was computed and $\Delta G_{bind}^{0}$ was finally determined (Table 1; Supplementary Table S1).

**TABLE 1:** Comparison between the numerically and the experimentally determined binding free energies for the FKBP12 + rapamycin ⇄ FKBP12•rapamycin reaction. MD results, i.e., the PMF and the corrected standard free energies, $\Delta G_{\mathrm{PMF}}$ and $\Delta G_{\mathrm{bind}}^{0}$ respectively, were obtained thanks to the CPUS approach. For each of the three variants, only the values corresponding to the lower-bound PMF profiles were selected (Joshi and Lin, 2019). Measurements are issued from a publication reporting on an inhibition assay in which the rotamase activity of FKBP12 was spectroscopically monitored using succinyl-AlaLeuProPhe-*para*-nitroalinide as a substrate and α-chimotrypsin digestion as a development reaction (DeCenzo et al., 1996b). These original data were acquired at 15°C, and we extrapolated them at 21.85°C, the temperature at which simulations were performed, so as to obtain the corrected binding free energies and equilibrium constants, $\Delta G_{\exp}^{\mathrm{corr}}$ and $K_{\exp}^{\mathrm{corr}}$ respectively (see Supplementary Table S2 and Supplementary Figure S8 for details).

FKBP12 variant	$\Delta G_{\mathrm{PMF}}$ (kcal/mol)	$\Delta G_{\mathrm{bind}}^{0}$ (kcal/mol)	$\Delta G_{\exp}^{\mathrm{corr}}$ (kcal/mol)	$K_{\exp}^{\mathrm{corr}}$ (nM)
WT	−11.09	−13.98	−12.53 ± 0.23	0.54 ± 0.21
Y82F	−10.21	−13.22	−11.98 ± 0.23	1.37 ± 0.50
D37	−8.08	−10.78	−9.61 ± 0.09	77.70 ± 11.97

### Curvilinear Path Tracing

The physical paths of dissociation were traced from the trajectories issued from the umbrella sampling simulations. First, for each run, the conformations were extracted at the 10-ps interval using the cpptraj module of AmberTools-20 and aligned with respect to FKBP12 only, the reference conformation being the one obtained after equilibration of the system. Doing so, the traversing of rapamycin from a bound to an unbound state could be plotted in the reference frame of the protein. Next, the aligned conformations were sorted with respect to the umbrella windows, which were sampled for 1 ns and thus contained a total of 100 conformations. For each window, the CoG of all 100 rapamycin molecules were computed and the geometric center of this ensemble (i.e., the window-center) was computed. The aligned conformation for which rapamycin was the nearest to this geometric center was chosen as the representative conformation for that umbrella window. All such umbrella window representative conformations were determined and their CoG, represented by colored spheres, used for tracing the physical path of dissociation. Finally, for each run, a black curve was drawn manually as a guide for the eye evidencing the separation process. Paths were only traced up to r = 10 Å, i.e., for the first 100 umbrella windows, so as to provide a clear vision of the initial steps of the dissociation reaction.

## Results

### Binding Free Energy Computations

All 10 ns-long unbiased MD simulations prior to CPUS showed root-mean-square deviations (RMSDs) within 1.0 Å and root-mean-square fluctuations (RMSFs) in the 0.4–3.6 Å range, which indicates the absence of any large conformational transition (Supplementary Figure S6). Thus, the FKBP12 variants in complexes with rapamycin are stable while well equilibrated. If this result is not surprising for the FKBP12_WT_•rapamycin complex, it also validates our simple modeling of FKBP12_Y82F_•rapamycin and FKBP12_D37V_•rapamycin.

Next, for each variant, 15 independent CPUS MD simulations were conducted and the corresponding PMF profiles were constructed. All curves flatten before reaching a CoG separation distance of 30.0 Å, which is a clear signature of complete dissociations (Figures 2A–C). The $\Delta G_{PMF}$ binding free energies could thus be identified as the last obtained PMF values. Additionally, in all cases, we could determine a cutoff separation distance at which the van der Waals interactions had decreased to zero (Supplementary Figure S7): $r_{*,cut}$ roughly ranges between 13 and 19 Å (Supplementary Table S1). With these cutoffs in hand we could further correct the $\Delta G_{PMF}$ according to Eq. 1, which yielded the $\Delta G_{bind}^{0}$ standard free energies (Supplementary Table S1). In application of the variational principle for each of the three complexes, we retained the lower-bound PMF profile for comparison with experimental data (Table 1, Figure 2D, Supplementary Figure S8, and Supplementary Table S2).

We first compared our simulations results with the data contained in the sole article we found that experimentally evaluated, with the same technique, the binding of rapamycin to all of the three FKBP12 variants hereby considered (Table 1) (DeCenzo et al., 1996b). With our CPUS approach, the differences of binding free energies between the mutant proteins and the wild-type one are $\Delta \Delta G_{bind}^{0}$ = 0.76 and 3.20 kcal/mol for Y82F and D37V, respectively. With the enzymatic inhibition assay reported in the literature, we have $\Delta \Delta G_{exp}^{corr}$ = 0.55 ± 0.33 and 2.92 ± 0.25 kcal/mol. Thus, both datasets are consistent if we consider uncertainties: We could predict *in silico* that Y82F is a nearly neutral mutation whereas D37V significantly weakens the interaction with rapamycin. Interestingly, the thermodynamic integration technique applied to the FKBP12**•**FK506 complex could also account for the very small destabilizing effect of Y82F, in similar agreement with experimental measurements (Pearlman and Connelly, 1995).

In a second step, we aimed at testing if our CPUS strategy can also provide reliable estimates of binding free energies (and not only of their differences). Therefore, we collected dissociation equilibrium constants for the FKBP12•rapamycin complex from a little less than 20 original research articles, spanning more than a 30 years-long period and describing measurements ranging from enzymatic inhibition assays to single-molecule ones, from isothermal calorimetry to surface plasmon resonance (Supplementary Table S2). The experimental data were first temperature corrected using published binding enthalpies (Supplementary Figure S8) (Connelly et al., 1993; Connelly et al., 1994) and then gathered in Figure 2D. The results that could be anticipated from Table 1 were confirmed: The *in silico* stability seems to be ≈1 kcal/mol higher than the one observed at the bench–although the reported values for FKBP12_WT_ are spread over more than 3 kcal/mol, the less negative binding free energies are possibly artifactual. Since the same discrepancy between simulations and measurements is observed for all three variants, the chances are high that it is a systematic effect. It could be due to the force-field inaccuracy and/or to the inability to fully account for the experimental system.

### Atom-Pair Distance Analysis

**FIGURE 3:**
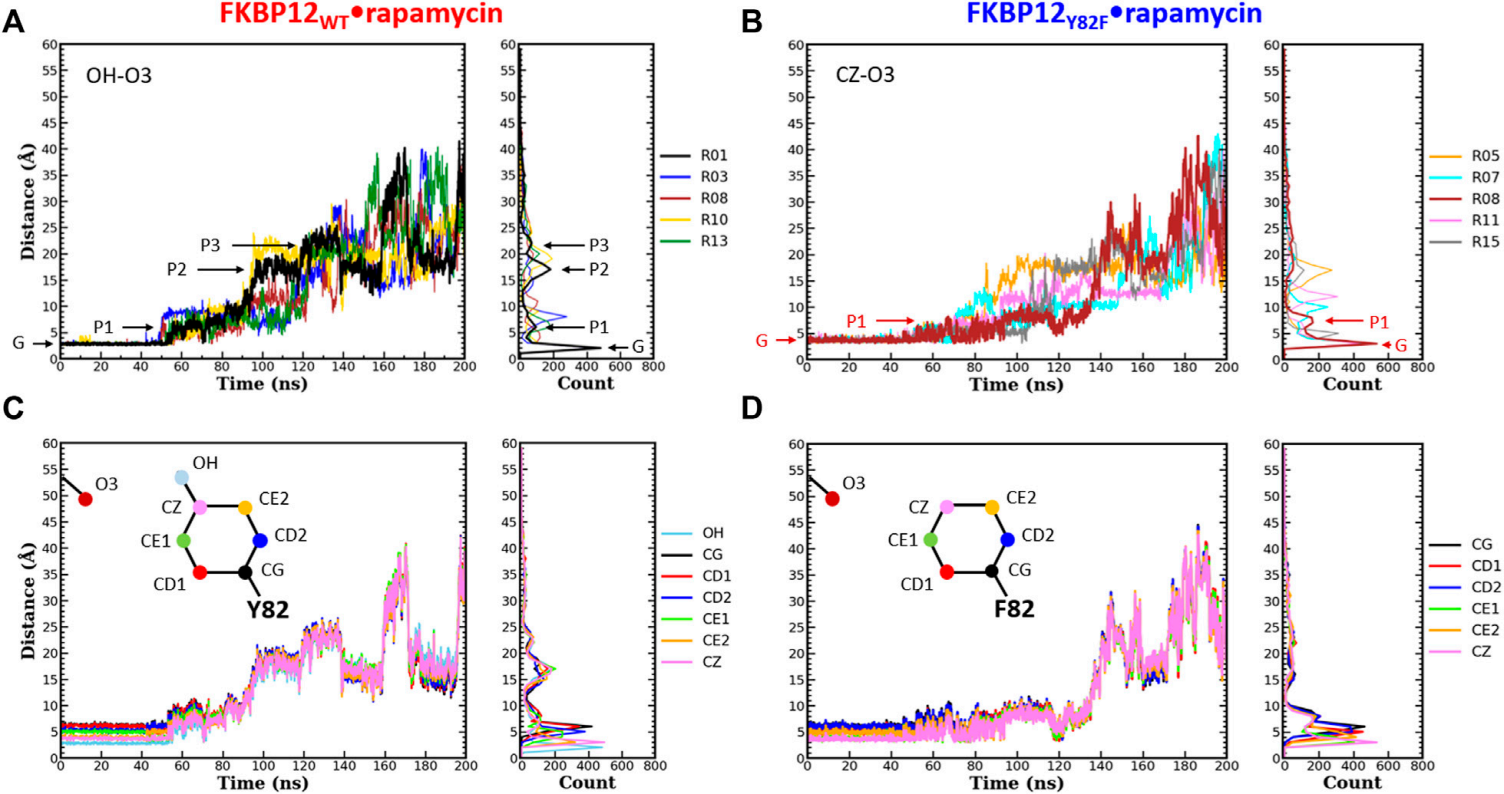
Comparison of the separation distance patterns obtained during the dissociation of rapamycin from the wild-type FKBP12 and from the Y82F mutant. Pulling on the molecular partners CoG starts at 10 ns. **(A)** Evolution of the distance between the O3 carbonyl oxygen of rapamycin and the OH hydroxyl oxygen of residue Y82 in the FKBP12_WT_•rapamycin complex. For the sake of clarity, data are only provided for the R01 lower-bound PMF profile and for four other ones. The G and Pi markers indicate, for the time-trace and the histogram associated with the lower-bound profile, the ground level and the different plateaus, respectively. The results for all 15 CPUS simulations are displayed in Supplementary Figures S9A. **(B)** Same plot for the distance between the O3 carbonyl oxygen of rapamycin and the CZ most distal carbon of residue F82 in the FKBP12_Y82F_•rapamycin complex. The lower-bound PMF profile is now R08, and the data for all 15 CPUS simulations are available in Supplementary Figures S9B. **(C)** Evolution of the distance between the O3 carbonyl oxygen of rapamycin and the seven heavy atoms of residue Y82 phenol group in the FKBP12_WT_•rapamycin complex. The data are only provided for the lower-bound PMF profile (see Supplementary Figures S9A for the whole set of curves). **(D)** Same plot for the distance between the O3 carbonyl oxygen of rapamycin and the six carbons of the residue F82 phenyl group in the FKBP12_Y82F_•rapamycin complex. Once more data are only provided for the lower-bound PMF profile, see Supplementary Figures S9B for an exhaustive presentation of the results.

**FIGURE 4:**
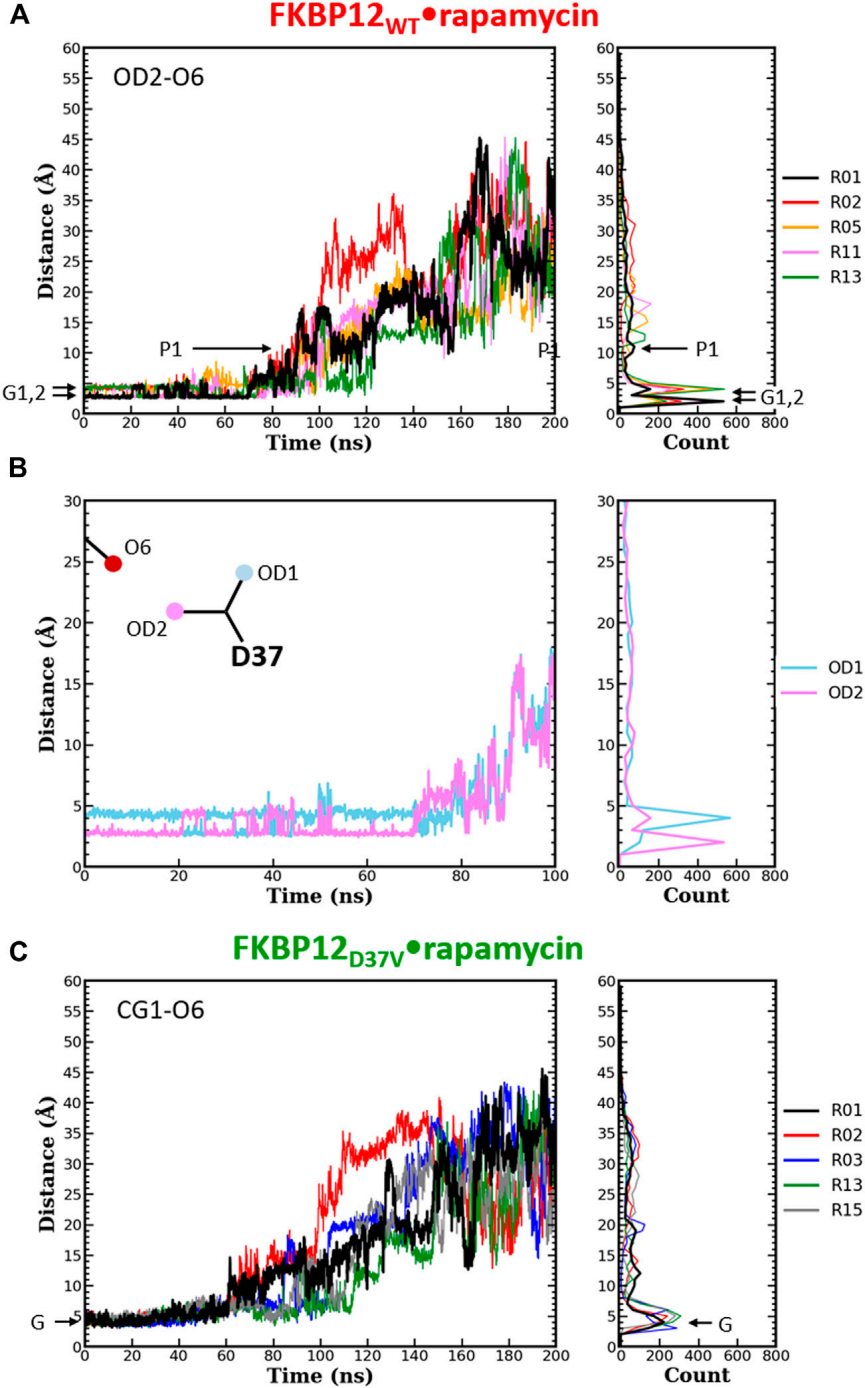
Comparison of the separation distance patterns obtained during the dissociation of rapamycin from the wild-type FKBP12 and from the D37V mutant. Pulling on the molecular partners CoG starts at 10 ns. **(A)** Evolution of the distance between the O6 hydroxyl oxygen of rapamycin and the OD2 carboxylate oxygen of residue D37 in the FKBP12_WT_•rapamycin complex. For the sake of clarity, the data are only provided for the R01 lower-bound PMF profile and for four other ones. The Gi and Pi markers indicate, for the time-trace and the histogram associated with the lower-bound profile, the ground levels and the different plateaus, respectively. The data for all 15 CPUS simulations are displayed in Supplementary Figures S10A. **(B)** Enlargement on the first tens of ns of the time-trace associated with the lower-bound PMF profile. The distance to the second carboxylate oxygen of residue D37 (OD1) has been added to evidence the H-bond switching between the two acceptor atoms. Supplementary Figures S10A provides similar data for the whole set of simulations. **(C)** Evolution of the distance between the O6 hydroxyl oxygen of rapamycin and one of the CG1 methyl carbon of residue V37 in the FKBP12_D37V_•rapamycin complex. Once more data are only provided for the R01 lower-bound PMF profile and for four other ones, see Supplementary Figures S10B for an exhaustive presentation of the results.

Even though both Y82 and D37 residues form a hydrogen bond with rapamycin, their role in ligand binding seems to differ: Removing the hydroxyl from the tyrosine has nearly no impact on the affinity (Connelly et al., 1994; Pearlman and Connelly, 1995; Kostrz et al., 2019) whereas exchanging the ethanoate side chain of the aspartate for an isopropyl one is significantly destabilizing (DeCenzo et al., 1996a; Singh et al., 2015; Kostrz et al., 2019). Since CPUS simulations could reproduce these energetic measurements, we next tried to see if the obtained trajectories could shed some light on the molecular mechanisms at work. More precisely, we performed a detailed analysis of both hydrogen bonded atom-pairs in the FKBP12_WT_•rapamycin complex, during the starting 10 ns-long unbiased simulations and during the following 190 ns-long CPUS ones (Figures 3, 4; Supplementary Figures S9, S10). Furthermore, the resulting patterns were compared with the ones obtained on the two mutant complexes, for atoms located at equivalent positions on the side chains.

During the first 50 ns simulating the wild-type complex (10 ns unbiased and 40 ns of CPUS), the length of the H-bond between Y82 and rapamycin remains unchanged, with ground level values around 3 Å for all 15 simulations. This fact is illustrated by the single position of the main peaks in the atom-pair distance histograms of Figure 3A and Supplementary Figure S9A (to illustrate our description different markers have been posted on the time-trace and on the histogram associated with the lower-bound PMF profile). Then, step-increases are observed and the atom-pair distances stabilize on plateaus located between 5 and 10 Å, depending on the simulation run. It thus yields secondary peaks with narrowly dispersed positions. As pulling progresses, these rapid transitions are sometimes followed by others, reflecting temporary interactions that last from ten to tens of ns and atom-pair distances that correspond to plateaus with higher average values. Such an evolution is evidenced by additional peaks in the histograms, peaks that are broader and that now lie in the 10 to 25 Å range. Incidentally, temporary back motions to a previous position are also possible, as exemplified by the R01 time-trace in Figure 3A. As one can see in Figure 3B and Supplementary Figure S9B, a similar behavior is observed for the carbon atom at the tip of the F82 phenyl ring in the FKBP12_Y82F_•rapamycin complex; the only differences are that the first step-increases occur roughly 5–10 ns earlier and that the starting atom-pair distance is 1–2 Å shifted (because we are one more covalent bond apart from the oxygen of the ligand). Such resembling stepwise dissociations for both the wild-type and the mutant proteins indicate that the aromaticity of the side-chain may play a role here. To probe this assumption, we plotted the atom-pair distance for all six carbons of the ring (Figures 3C,D and Supplementary Figure S9) and indeed evidenced a concerted motion making us think that a persistent, long-range pseudo-bond could be relevant to anion-π interactions between the side-chain benzyl ring and the oxygen atoms of the rapamycin (Schottel et al., 2008; Chifotides and Dunbar, 2013; Giese et al., 2016; Lucas et al., 2016).

The same detailed approach was subsequently applied to study the role of D37 in rapamycin binding. In the wild-type complex, the length of the H-bond between this residue and the ligand remains nearly unchanged during the first 50–70 ns of simulation: It just fluctuates between 2.7 and 4.0 Å (Figure 4A and Supplementary Figures S10A). The origin of this fluctuation is clearly revealed when considering the other oxygen of the aspartate side-chain (Figure 4B and Supplementary Figures S10A): The two heteroatoms alternatively interact with rapamycin upon rotation of the carboxylate group, which results in two sharp peaks in the atom-pair distance histograms. Once the H-bond is broken, the oxygens of D37 and the one of the macrolide are torn in a continuous manner, showing very little step-like distance increments. Moreover, if a plateau is observed, it manifests itself in the histogram through a low and shallow peak, i.e., a somehow short and weak interaction. Comparatively, in the FKBP12_D37V_•rapamycin complex the methyl groups of valine do not display any switching (Figure 4C and Supplementary Figures S10B). Furthermore, all peaks in the histograms are broader than in the wild-type case, the one corresponding to the complex at equilibrium as well as the ones corresponding to transient interactions occurring upon dissociation. These two observations are in line with the inability of the alkyl moieties to form strong, directional intermolecular bonds. Finally, the most obvious signature of the weakening of the association between rapamycin and FKBP12 after the D37V substitution is that the atom-pair distances start to increase earlier, nearly as soon as the bias is applied.

### Dissociation Path Tracing

The umbrella sampling simulation trajectories were processed to trace the physical paths of dissociation for the FKBP12_WT_•rapamycin, FKBP12_Y82F_•rapamycin, and FKBP12_D37V_•rapamycin complexes (Supplementary Figures S11–S13, respectively). Rapamycin is always seen to traverse to the solvent in a curvilinear way, exiting through either the top left or the top right of the binding cavity. For the paths corresponding to the lower-bound PMF profiles, the macrolide initially slides on the surface of the protein smoothly and then wind a lot. In contrast, the paths associated with the highest PMF profiles are relatively less curvy (Supplementary Figure S14). The highly curved nature of lower-bound paths suggests that rapamycin does not get stuck on FKBP12 and tends to free from its surface towards the solvent quite early, or at least it does not get trapped into local energy minima. On the other hand, the highest PMF paths for FKBP12_WT_ and FKBP12_Y82F_ display accumulations of spheres that could be the signature of some interactions outside of the binding site.

## Discussion and Conclusions

In this work, we present a curvilinear-path umbrella sampling simulation approach to estimating the free energy of binding for the FKBP12•rapamycin complex. The strategy consists of several crucial steps that includes sampling enhancement along multiple independent curvilinear paths, construction of PMFs, determination of the lower-bound profile and of the corresponding $\Delta G_{PMF}$ term, and then correction of this term to obtain the standard free energy of binding. The corresponding theoretical framework was recently developed and successfully implemented to estimate protein–protein interaction energetics (Joshi and Lin, 2019). Here, we extended the approach to a protein–ligand system, and our results are in good agreement with reported experimental data. Although overall the CPUS approach is quite effective to estimate reliably interaction energetics, there are a few things one needs to keep in mind before computing the correction term. The umbrella implementation needs to decide the distance range, i.e., starting reference bound distance and the end separation distance. Upon constructing PMF, the profile may depict a signature of complete dissociation in terms of converging of the curve to some value. However, in the computation of the correction term, we suggested interaction energetics (VDW component) criterion to judge the complete dissociation (see the Methods and Materials section). The simulations should be run to that separation distance where the VDW component converges to zero. In the case of protein–small ligand dissociation reaction, this distance may vary a bit depending upon the path that the ligand could take. Therefore, while setting the end separation distance, it is advised to set it to sufficiently large values, considering that the dissociating compound may slide/rebind on the protein surface after an initial dissociation.

Although Lee and Olson mentioned the necessity of sampling along a curvilinear physical trajectory, they estimated the absolute binding affinities using a vectorial path (Lee and Olson, 2006). Furthermore, their energetic results were in good agreement with the experiments but quadratic approximations were required. Doudou et al. (2009) discussed rigorously the problem associated with energetics obtained by sampling along a predefined vectorial reaction coordinate and pointed out related inconsistencies in the free energy estimation along different linear paths/directions. The CPUS approach is a naïve approach that tries to address these issues by conducting successive umbrella sampling simulations. The approach is applicable especially when a precise input, such as the detailed shape of the binding pocket or a predefined vector of confinement, is unavailable. It relies on the minimal use of restraint potentials (namely, only one biasing potential in the umbrella sampling simulation); hence, it fairly bypasses some major challenges one can face for appropriate de-biasing. However, the cost has to be paid in terms of conducting a sufficiently large number of umbrella sampling simulations to find the lower-bound of the PMF profiles. In addition, due to the complex nature of biomolecular systems it is rather difficult, if not impossible, to decide *a priori* the number of multiple-walker umbrella sampling simulations to be conducted. This difficulty is actually also shared by any variational-based approaches, e.g., quantum Monte Carlo (Ceperley, 1978; Ceperley and Alder, 1980). It would be always a good sign to find some robust lower-bound from the multiple PMF profiles. Thus, the current work delivers an important message that the PMF profile from any single umbrella sampling simulation (irrespective of whether predefined vector-based or non-predefined curved-path-based sampling enhancement) may likely be misleading. With ongoing advances in GPU architecture, the simulation time is expected to keep decreasing, enabling more simulations to be performed in parallel. Indeed, the implicit/continuum solvent model would rather be a more natural choice for rapid estimation and reducing the computation cost. However, some serious concerns are yet to be suitably resolved, such as too large numerical ranges of estimated energies and strong dependence of the energetics on the employed continuum solvation (such as the choice of protein dielectric constant, the definition of protein boundary, etc.) (Genheden and Ryde, 2015). Therefore, explicit solvent modes are highly preferred.

A related issue in the CPUS approach is to evaluate if the molecular behaviors associated with the lower-bound PMF profile significantly differ from the ones associated with the higher profiles. In our previous work, we traced the protein–protein dissociation paths and observed that there was a clear difference in the direction of traverse between the lower-bound case and the rest (Joshi and Lin, 2019). This distinction between the paths was made possible because the two prominent paths had been previously evidenced using an extensive brute force adaptive MD simulation approach (Plattner et al., 2017). In the case of the FKBP12•rapamycin system, we observed that the rapamycin initially moves up and is then released in the solvent by taking either a left or a right path (Supplementary Figures S11–S14). However, we could not find any supportive correlation between the plateau value of the PMF profile and the path taken. Similarly, we looked for correlations between the PMF final value and the presence of sphere clusters along the way to dissociation, the latter signature being interpreted as the temporary immobilization of rapamycin in a local energy minimum present at the surface of FKBP12. Although few of such potential wells could be identified (see, for instance, the red circles in Supplementary Figure S14), these finding are at the moment only qualitative. As a consequence, the physical paths of dissociation do not enable us to differentiate one or a subset of simulations from the other ones. In fact, it would be interesting to see whether the paths traced from the CPUS approach are consistent with the ones revealed by other pathway sampling methods, such as the string method (Weinan et al., 2002), the minimum free energy path (Maragliano et al., 2006), the adaptive and unconstrained enhanced sampling (Miao and McCammon, 2016), the nudged elastic band (NEB) method (Jónsson et al., 1998), or stochastic difference equation (in length version) SDEL (Arora and Schlick, 2005).

In this work, we also performed a detailed analysis on the atom-pair distance distribution patterns (Figures 3, 4 and Supplementary Figures S9, S10). For all three variants the data that yielded the lower-bound PMF profile and the ones issued from the 14 other runs were rather similar in terms of global behavior, i.e., notwithstanding the variability one can expect when looking at single molecules. Since the ligand conformation may also play a crucial role in the dissociation mechanism, we plotted the temporal evolution of the RMSD for the heavy atoms (C, N, O) of rapamycin (Supplementary Figures S15–S17). Although some peaks or steps are present, the overall RMSD profiles are quite stable for all CPUS simulations and the dissociation process does not seem to be coupled with important conformational transitions in the macrolide. The two hydrogen-bonded atom-pairs investigated here, i.e., Y(F)82-O3 and residue D(V)37-O6, have significantly different patterns. Moreover, the comparison of distance distribution patters between atom-pairs of the side-chain benzyl ring of FKBP12 variants (Y82 and F82) and the O6 of rapamycin pointed out that they might be involved in the anion-π type of interactions. These atomistic-level factors could contribute to the excess dissipation along the curvilinear trajectory of dissociation, and further detailed investigations are needed to have a comprehensive understanding of the relationship between estimated energetics and atom-pair interactions.

Thus, the CPUS approach paves the way to reveal binding energetics along with mechanistic details. The approach is highly generalized and can be implemented to almost any protein–ligand systems, along with protein–protein systems, as well. The approach offers a strong platform to perform several types of in-depth analysis towards revealing the underlying mechanistic details. The drug discovery process often deals with known drug targets and with new or modified drug molecules. In the case of rapamycin-FRB interactions, one such study recently identified DL001 compound that reduces the side effects *in vivo* (Schreiber et al., 2019). The CPUS approach-based characterization could be helpful for further investigation and for the design of such effective drugs.

## Data Availability

The raw data supporting the conclusions of this article will be made available by the authors, without undue reservation.
