# Supplementary material for: A Curvilinear-Path Umbrella Sampling Approach to Characterizing the Interactions Between Rapamycin and Three FKBP12 Variants
Source: Front Mol Biosci. 2022 Jul 8;9:879000. doi: 10.3389/fmolb.2022.879000 (PMC9304761; doi:10.3389/fmolb.2022.879000)
Supplement: Supplementary file 1 [file DataSheet1.pdf]

Supplementary Material

A Curvilinear-Path Umbrella Sampling Approach to Characterizing the Interactions Between Rapamycin and Three FKBP12 Variants

Dhananjay C. Joshi[1], Charlie Gosse[2], Shu-Yu Huang[1], Jung-Hsin Lin[1,3,4,5,6] *

[1] Research Center for Applied Sciences, Academia Sinica, Taipei, Taiwan

[2] Institut de Biologie de l'Ecole Normale Supérieure, ENS, CNRS, INSERM, PSL Research University, Paris, France

[3] Institute of Biomedical Sciences, Academia Sinica, Taipei, Taiwan

[4] Biomedical Translation Research Center, National Biotechnology Research Park, Academia Sinica, Taipei, Taiwan

[5] School of Pharmacy, College of Medicine, National Taiwan University, Taipei, Taiwan

[6] College of Engineering Sciences, Chang Gung University, Taipei, Taiwan

***** jhlin@gate.sinica.edu.tw

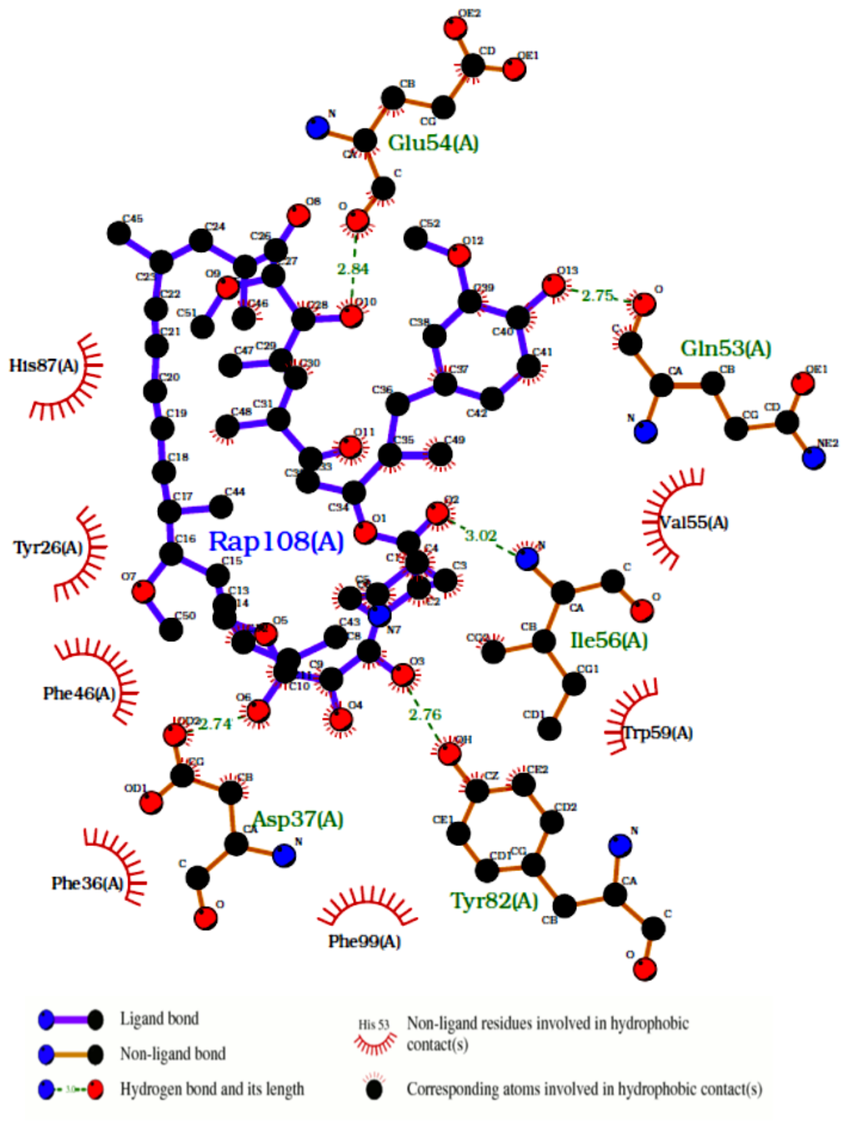

SUPPLEMENTARY FIGURE S1 | LIGPLOT representation[1] of the interactions between FKBP12$_{WT}$ and rapamycin, based on crystal structure PDB 1FKB.[2]

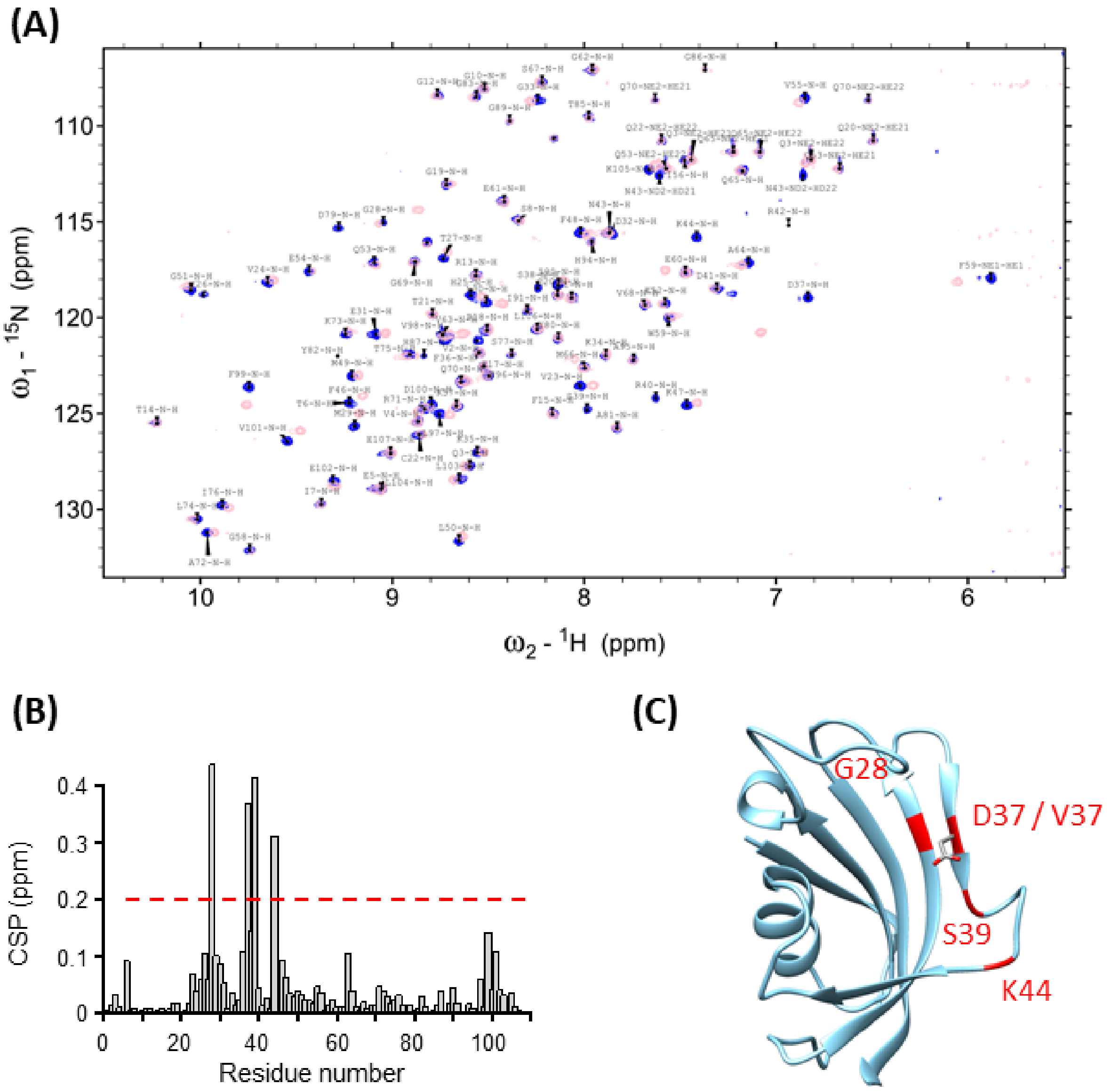

SUPPLEMENTARY FIGURE S2 | Comparison between the electronic environment of the amide NH bounds in FKBP12$_{WT}$ and FKBP12$_{D37V}$. **(A)** ^{1}H-^{15}N hetero-nuclear single quantum coherence (HSQC) NMR spectra for the wild-type protein (blue) and for the D37V mutant (pink). Measurements were performed at 298 K on a 600 MHz NMR spectrometer (UltraShield 600 and AVANCE 600 system, Bruker). ^{15}N-labeled proteins were purified using protocols adapted from the literature[3] and dissolved at 50 µM in a buffer containing 20 mM sodium phosphate pH 7.0 and 100 mM NaCl. **(B)** Corresponding analysis of the chemical shift perturbations,[4] computed as $\mathbf{CSP} = \sqrt{(\Delta\boldsymbol{\delta H})^2 + (\mathbf{0.154} \times \Delta\boldsymbol{\delta N})^2}$ with $\Delta\boldsymbol{\delta H}$ and $\Delta\boldsymbol{\delta N}$ being the respective proton and nitrogen chemical shift difference between the wild-type protein and the mutant one.[5] **(C)** Structure of FKBP12$_{WT}$ on which the residues highlighted in red are the ones that have a CSP larger than the mean plus two standard deviation upon substitution of D37 by V37,[4] i.e. $\langle \boldsymbol{CSP} \rangle + \mathbf{2}\boldsymbol{\sigma}_{\boldsymbol{CSP}}$ = 0.198 ppm.

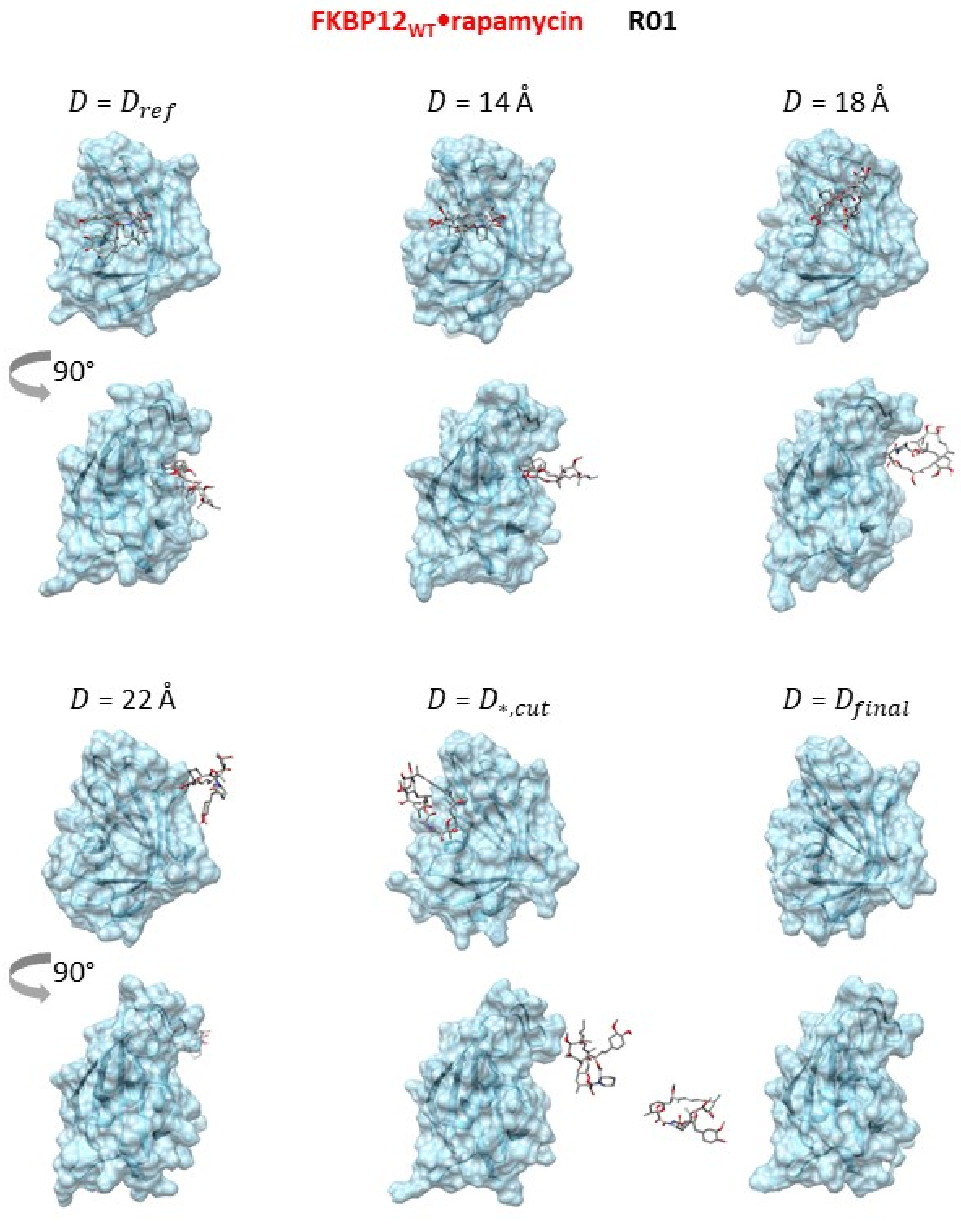

SUPPLEMENTARY FIGURE S3 | Snapshots taken during the dissociation of rapamycin from FKBP12$_{WT}$ for the MD CPUS run resulting in the lower-bound PMF profile highlighted in **Figure 2A**.

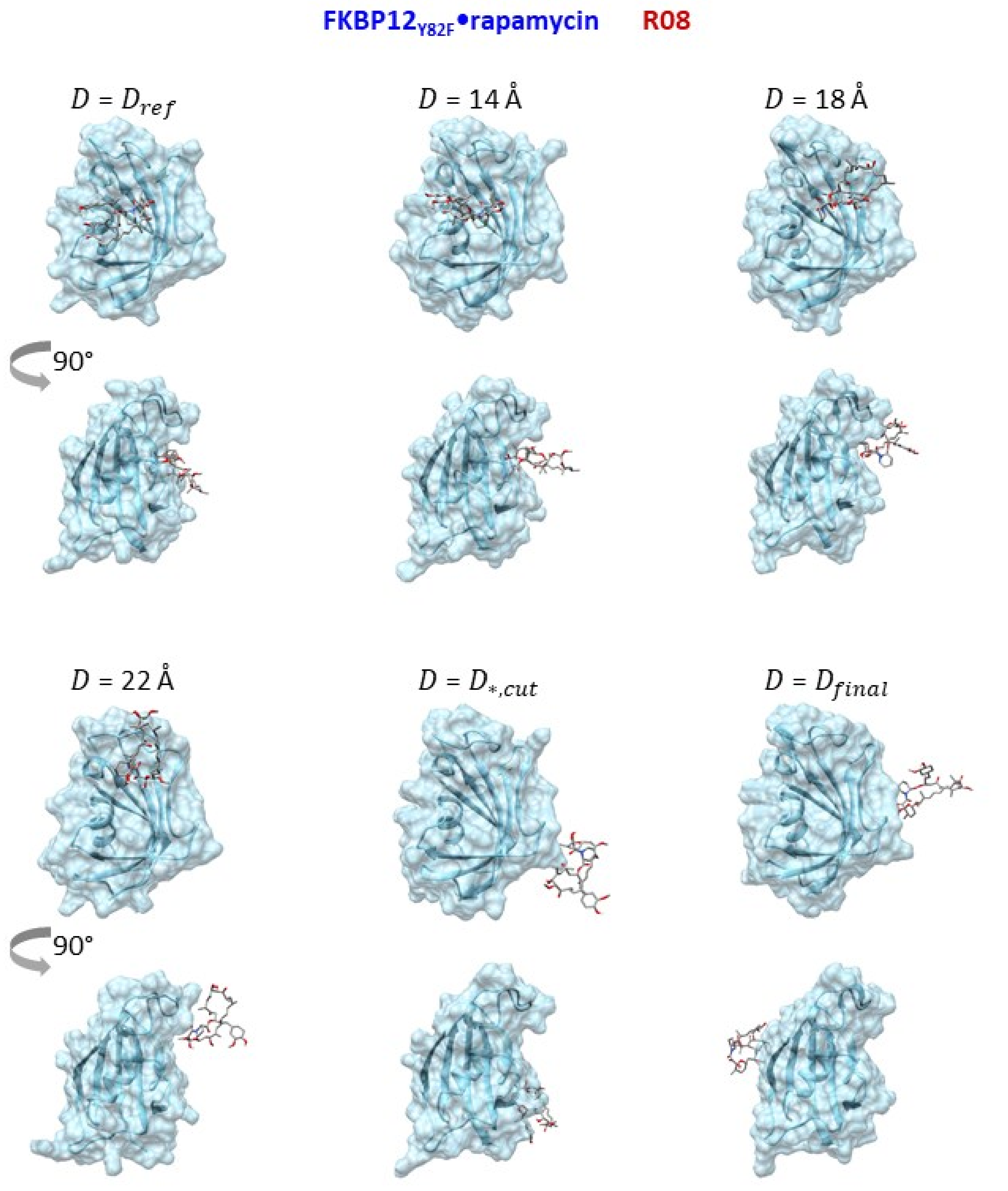

SUPPLEMENTARY FIGURE S4 | Snapshots taken during the dissociation of rapamycin from FKBP12$_{Y82F}$ for the MD CPUS run resulting in the lower-bound PMF profile highlighted in **Figure 2B**.

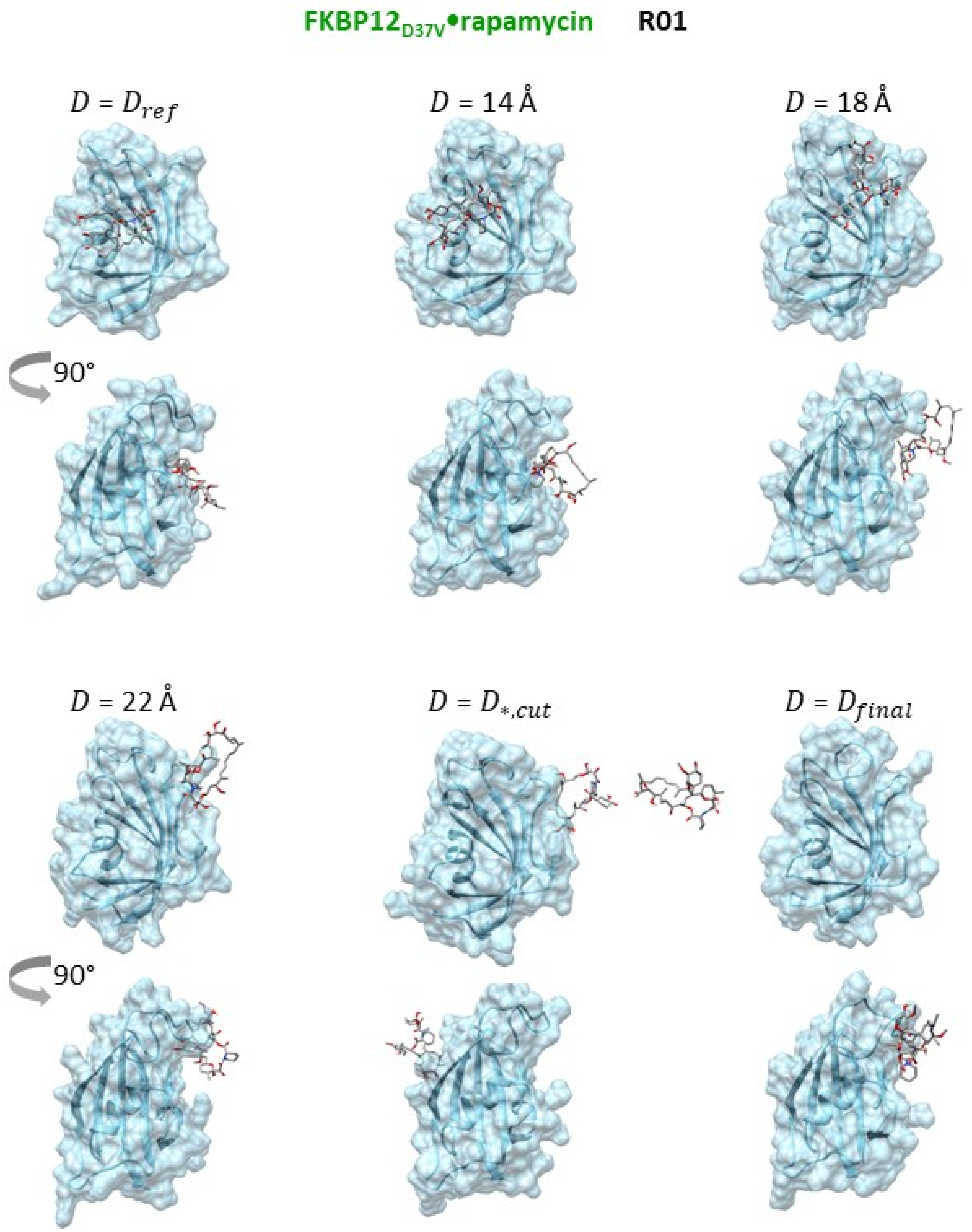

SUPPLEMENTARY FIGURE S5 | Snapshots taken during the dissociation of rapamycin from FKBP12$_{D37V}$ for the MD CPUS run resulting in the lower-bound PMF profile highlighted in **Figure 2C**.

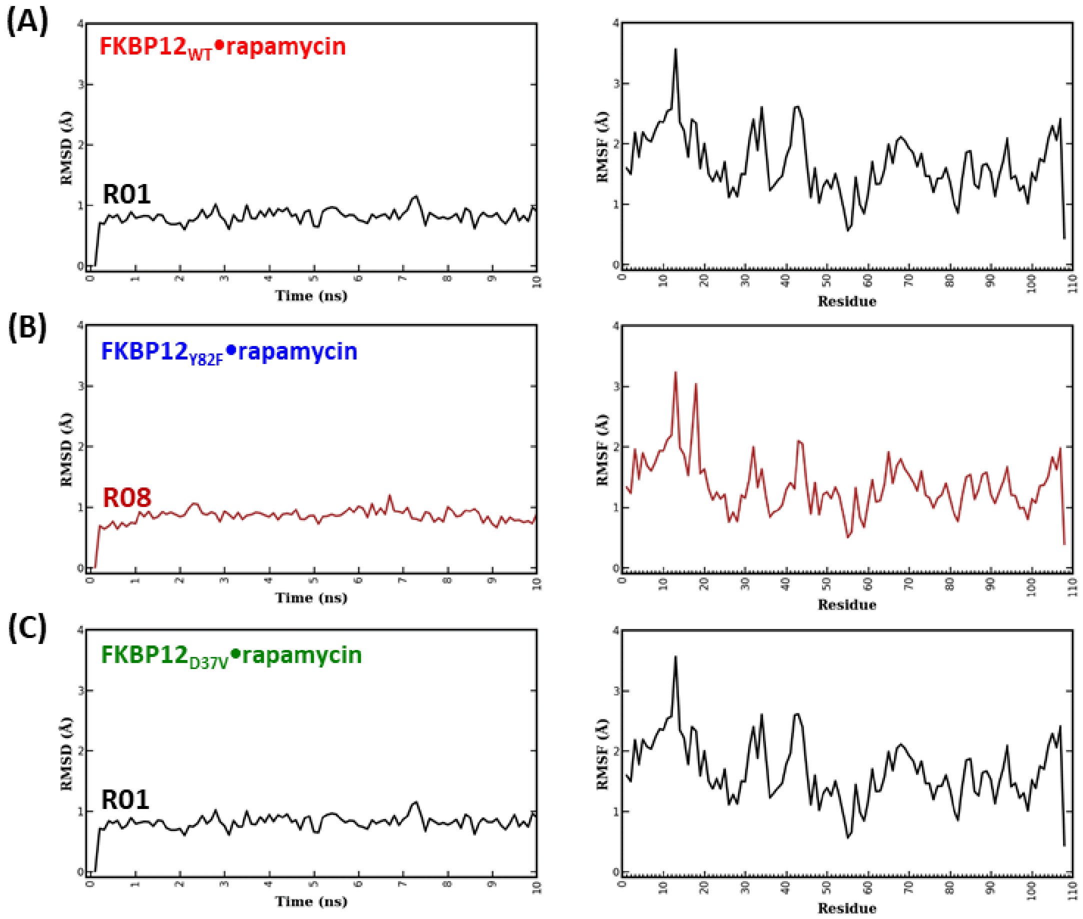

SUPPLEMENTARY FIGURE S6 | Stability assessment for the complexes formed between rapamycin and the three FKBP12 variants. For each protein the 10 ns-long unbiased MD simulations realized prior to CPUS were analyzed and as representative examples are here displayed the results obtained for the 3 runs that will lead to the lower-bound PMF profiles highlighted in **Figure 2** – similar plots are obtained for all the other 42 runs (not shown). **(A)** Reference data for the $FKBP12_{WT}$•rapamycin complex. The root-mean-square deviation (RMSD) with respect with the starting configuration was computed on all Cα atoms. The root-mean-square fluctuation (RMSF) was computed by residue. **(B)** Same data for $FKBP12_{Y82F}$•rapamycin. **(C)** Same data for $FKBP12_{D37V}$•rapamycin.

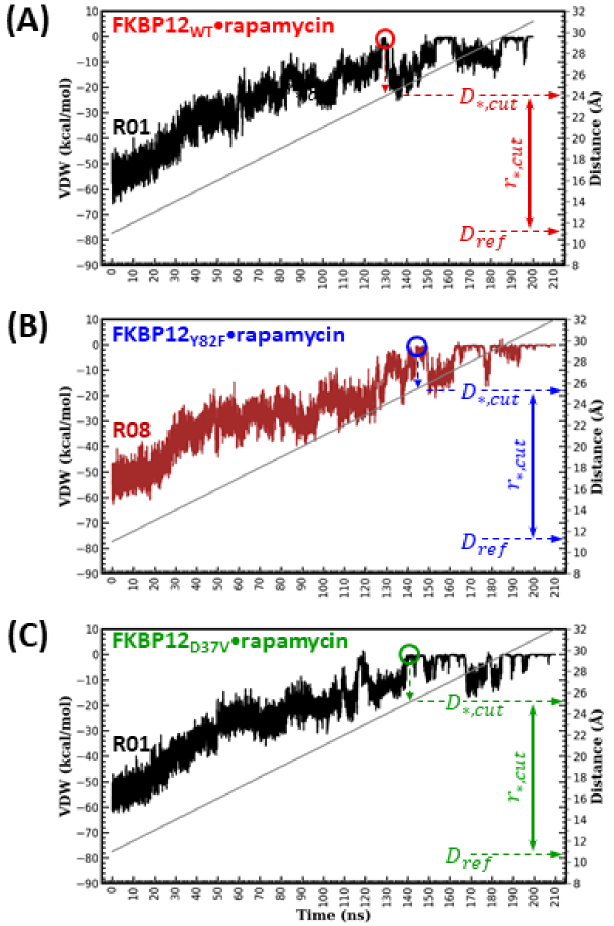

SUPPLEMENTARY FIGURE S7 | Van der Waals component of the interaction energy, WDV, as a function of the CPUS MD simulation time for the 3 runs resulting in the lower-bound PMF profiles highlighted in **Figure 2** – similar plots are obtained for all the other 42 runs (not shown). **(A)** Data for the FKBP12$_{WT}$•rapamycin complex. The time at which VDW cancels is converted in CoG distance D thanks to the linear relationship between those two parameters (grey diagonal). Then, the cutoff separation distance is computed by simply subtracting to the obtained value the mean CoG distance in the complex at equilibrium: $r_{*,cut} = D_{*,cut} - D_{ref}$. **(B)** Same data for FKBP12$_{Y82F}$•rapamycin. **(C)** Same data for FKBP12$_{D37V}$•rapamycin.

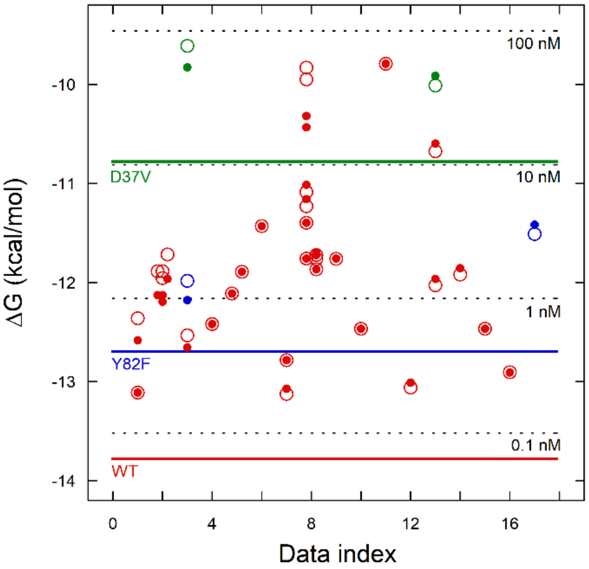

SUPPLEMENTRARY FIGURE S8 | Effect of the temperature correction on the experimentally determined binding free energies for the FKBP12 + rapamycin ⇄ FKBP12•rapamycin reaction. Full circles correspond to ΔG_{exp}^{meas}, the measurements retrieved from the literature and performed at various temperatures T^{meas}, whereas open circles correspond to ΔG_{exp}^{corr}, the same data extrapolated at T^{sim} = 21.85 °C, the temperature at which MD simulations were run (see **Supplementary Table S2** for numerical values, for indexing, and for details on the correction procedure). Red markers refer to the wild-type FKBP12, blue ones to the Y82F mutant, and green ones to the D37V mutant. Data have been sorted along the x-axis according to their publication year; moreover, to emphasize on possible biases due to individual practises we have clustered together all measurements coming from a same laboratory. The three colored lines correspond to ΔG_{bind}^{0}, the MD results obtained thanks to the CPUS approach (see **Table 1** for numerical values). The four black and dashed lines indicate the ΔG values associated with the 0.1, 1, 10, and 100 nM dissociation equilibrium constants.

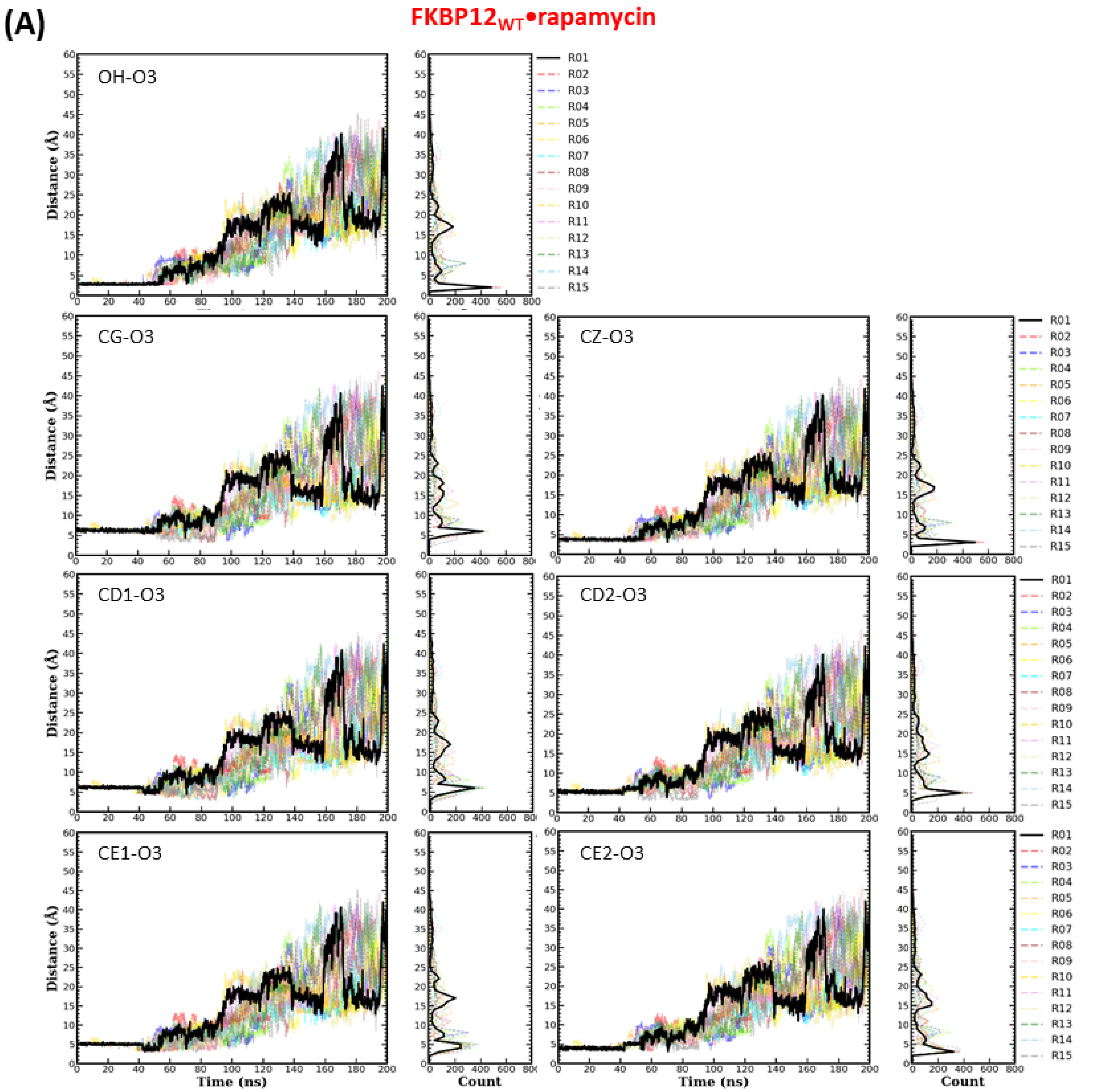
(A)
FKBP12$_{WT}$•rapamycin
OH-O3
CG-O3
CZ-O3
CD1-O3
CD2-O3
CE1-O3
CE2-O3
Distance (Å)
Time (ns)
Count
R01
R02
R03
R04
R05
R06
R07
R08
R09
R10
R11
R12
R13
R14
R15

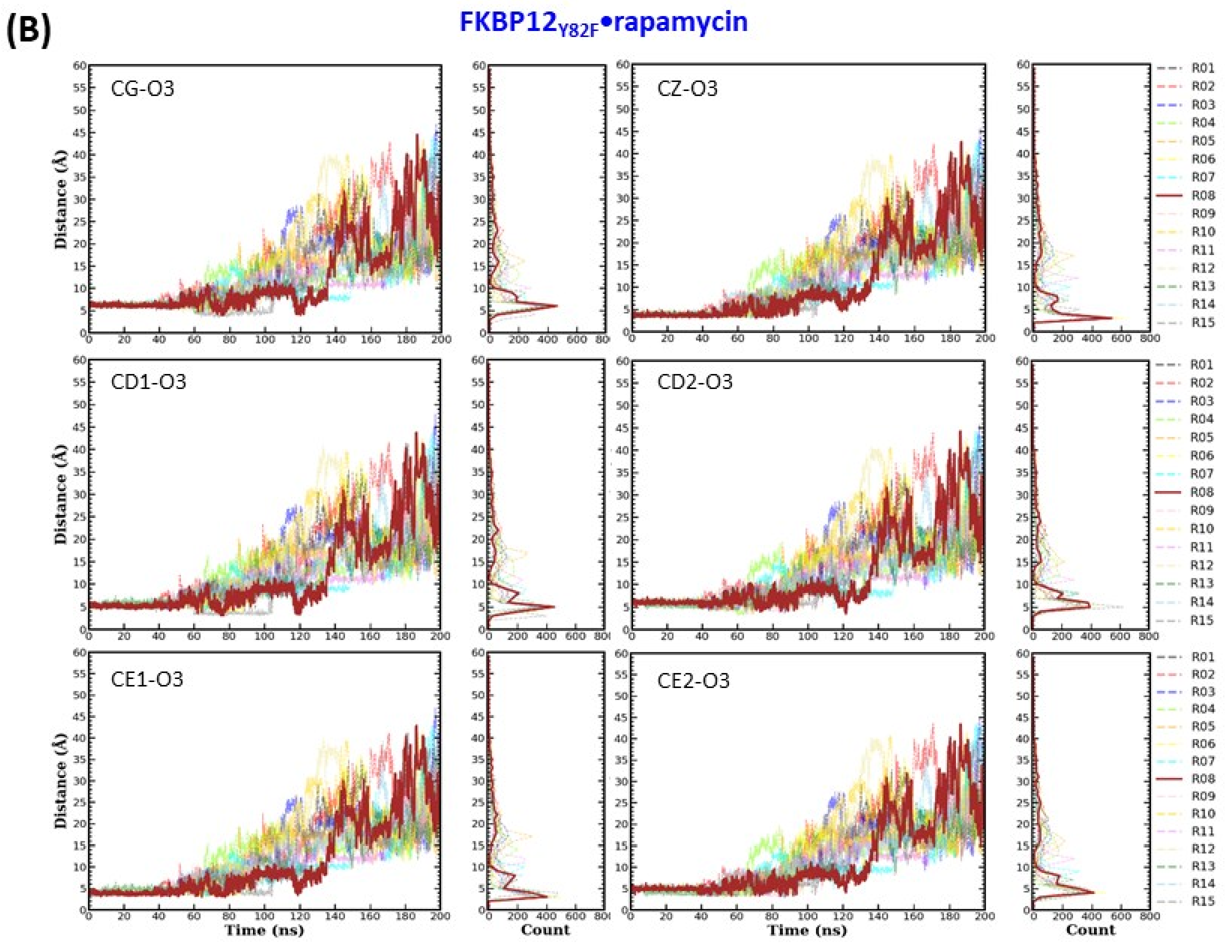

SUPPLEMENTARY FIGURE S9 | Comparison of the separation distance patterns obtained during the dissociation of rapamycin from the wild-type FKBP12 and from the Y82F mutant. Pulling on the molecular partners CoG starts at 10 ns. **(A)** Evolution of the distance between the O3 carbonyl oxygen of rapamycin and the oxygen as well as the six carbons of residue Y82 phenol group in the $FKBP12_{WT}$•rapamycin complex. Data are provided for all 15 runs and the one associated with the lower-bound PMF profile is in a brighter color. **(B)** Same plots for the distance between the O3 carbonyl oxygen of rapamycin and the six carbons of residue F82 phenyl group in the $FKBP12_{Y82F}$•rapamycin complex.

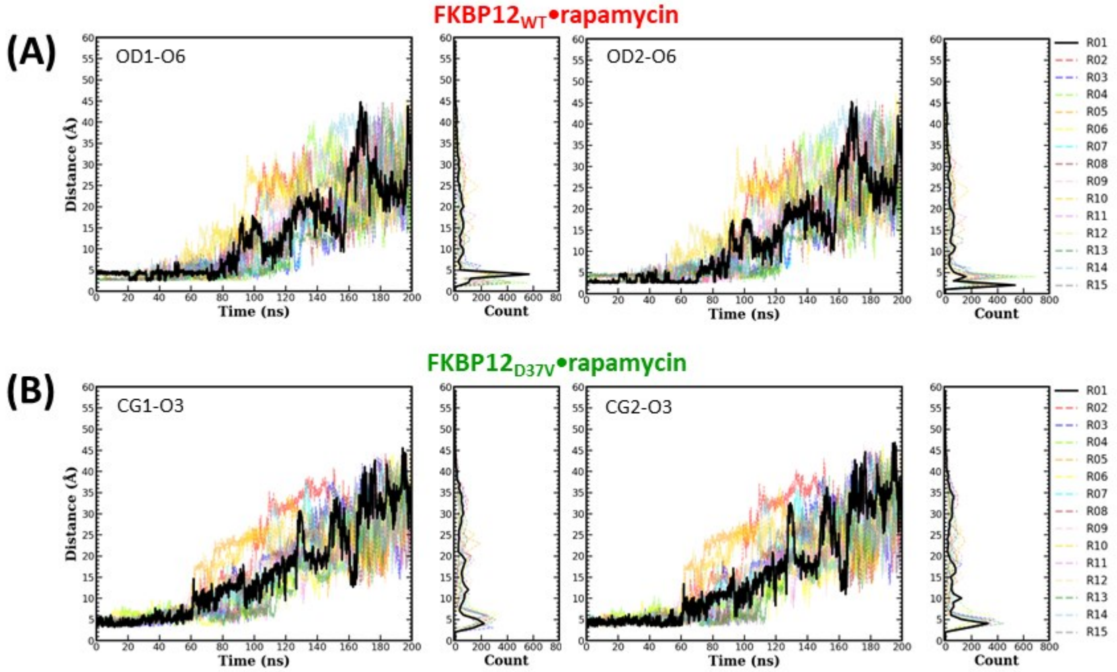

SUPPLEMENTARY FIGURE S10 | Comparison of the separation distance patterns obtained during the dissociation of rapamycin from the wild-type FKBP12 and from the D37V mutant. Pulling on the molecular partners CoG starts at 10 ns. **(A)** Evolution of the distance between the O6 hydroxyl oxygen of rapamycin and the 2 oxygen of residue D37 carboxyl group in the $FKBP12_{WT}$•rapamycin complex. Data are provided for all 15 runs and the one associated with the lower-bound PMF profile is in a brighter color. **(B)** Same plots for the distance between the O6 hydroxyl oxygen of rapamycin and the 2 carbons of residue V37 methyl groups in the $FKBP12_{D37V}$•rapamycin complex.

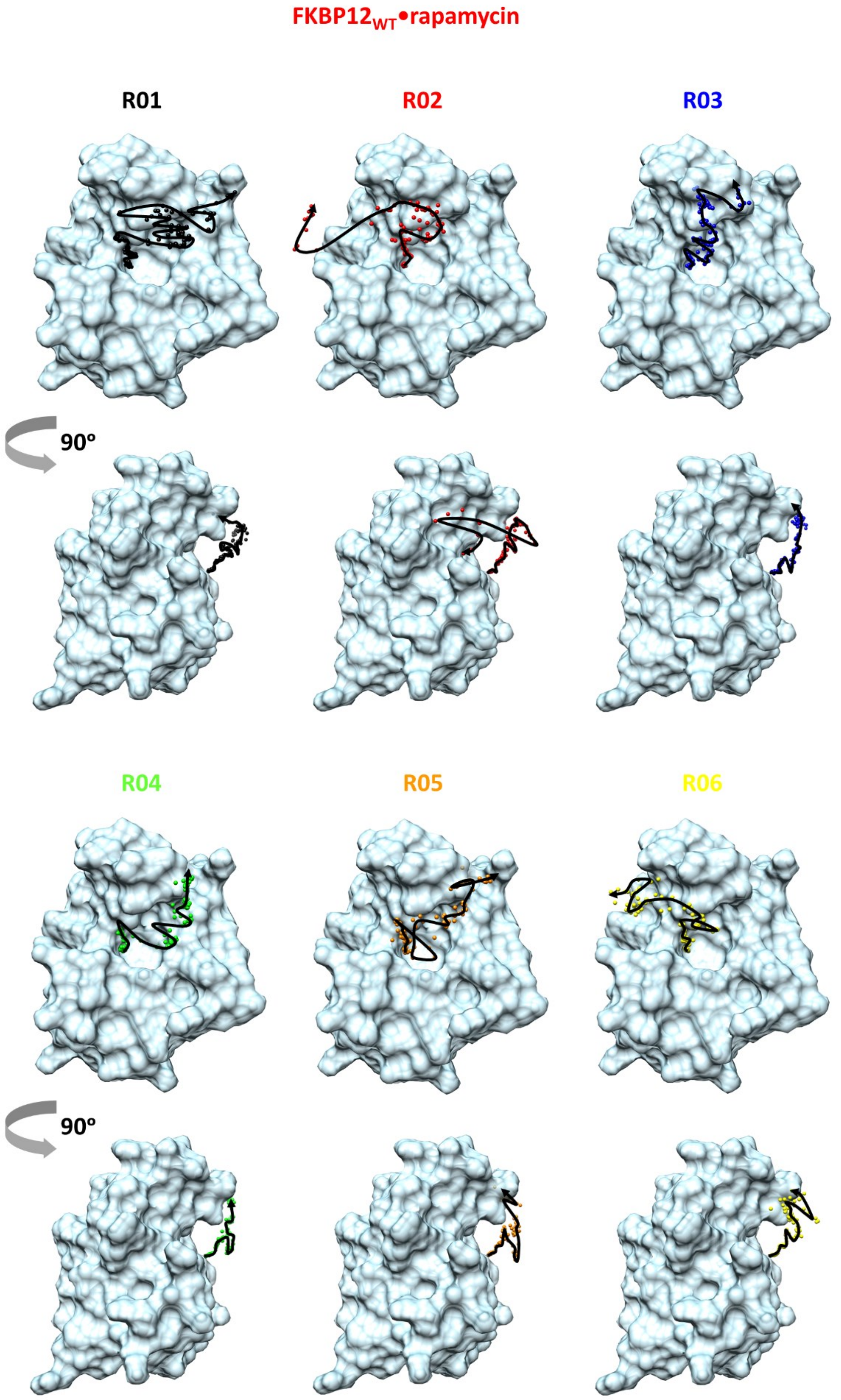
FKBP12$_{WT}$•rapamycin
R01
R02
R03
90º
R04
R05
R06
90º

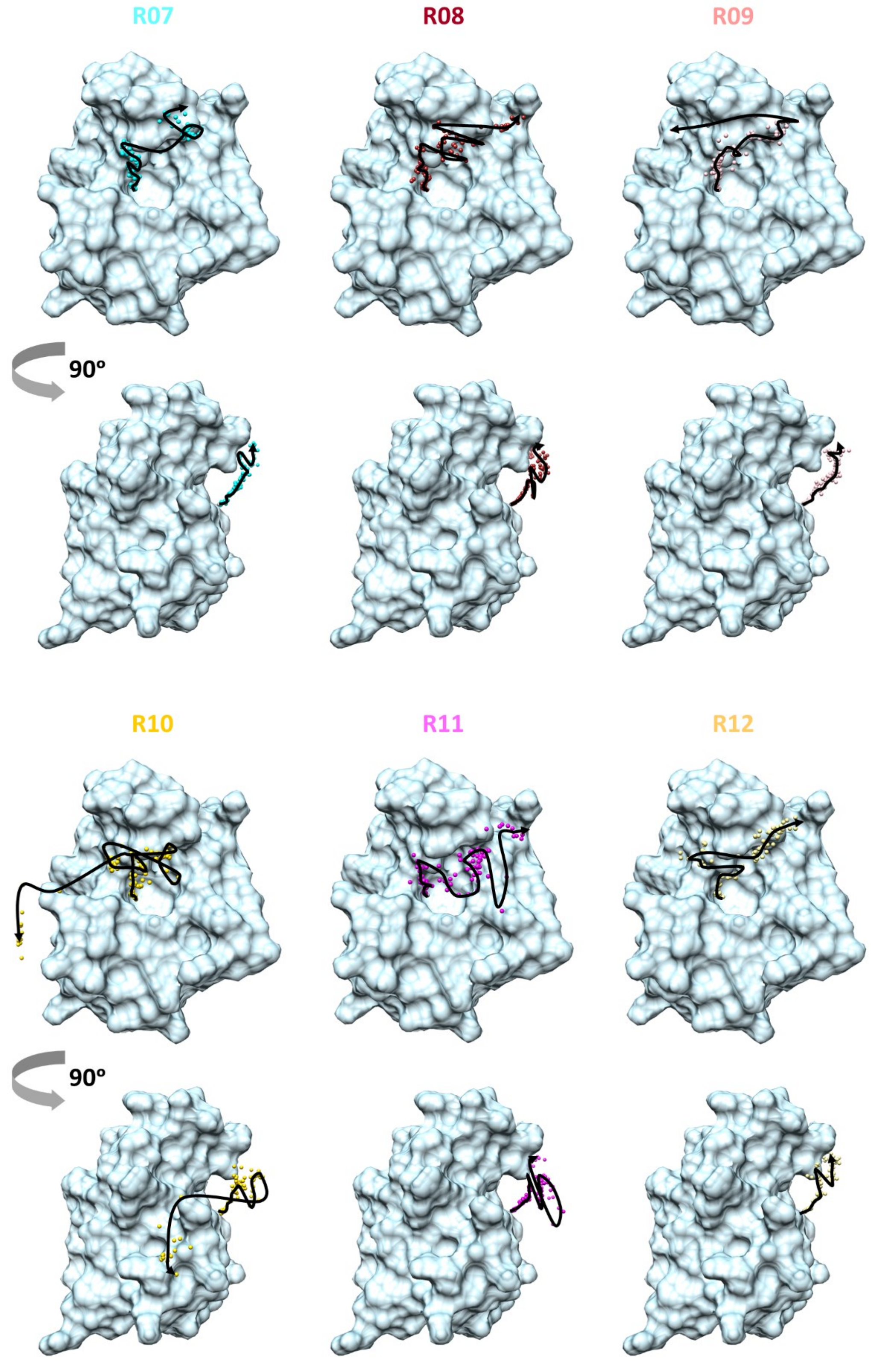
R07
R08
R09
90º
R10
R11
R12
90º

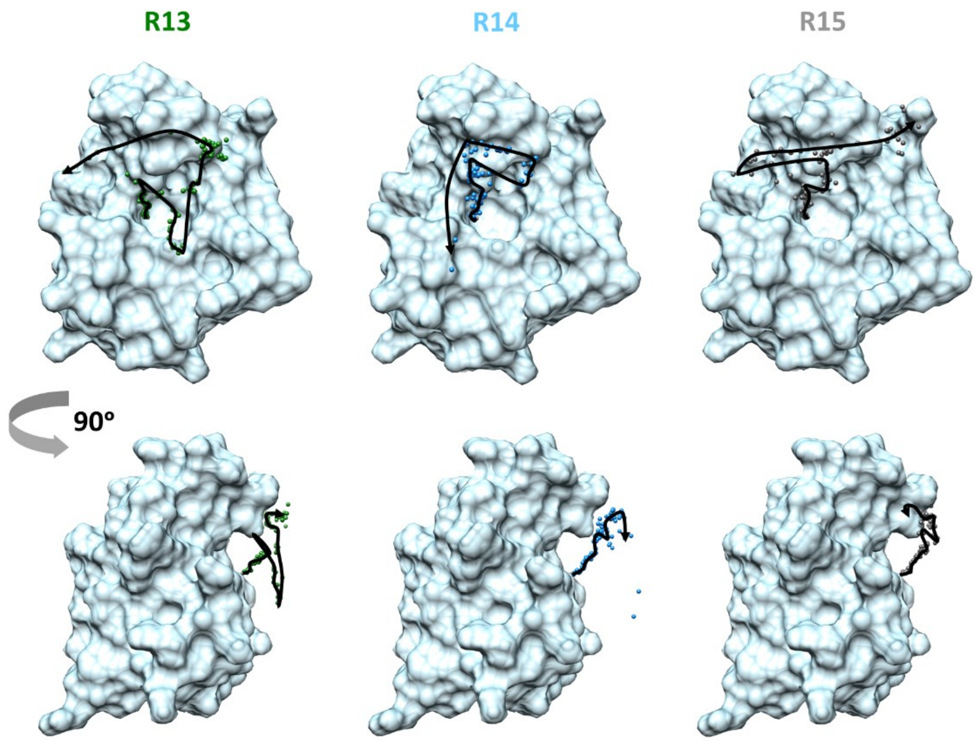

SUPPLEMENTARY FIGURE S11 | Curvilinear paths obtained for the $FKBP12_{WT}$•rapamycin → $FKBP12_{WT}$ + rapamycin dissociation. Each sphere roughly corresponds to the barycenter of all rapamycin CoG extracted from one umbrella sampling window of CPUS MD simulations. It has the same color than the PMF profile it is associated with. The black lines are guides for the eye. Data are only displayed up to $\boldsymbol{r}$ = 10 Å.

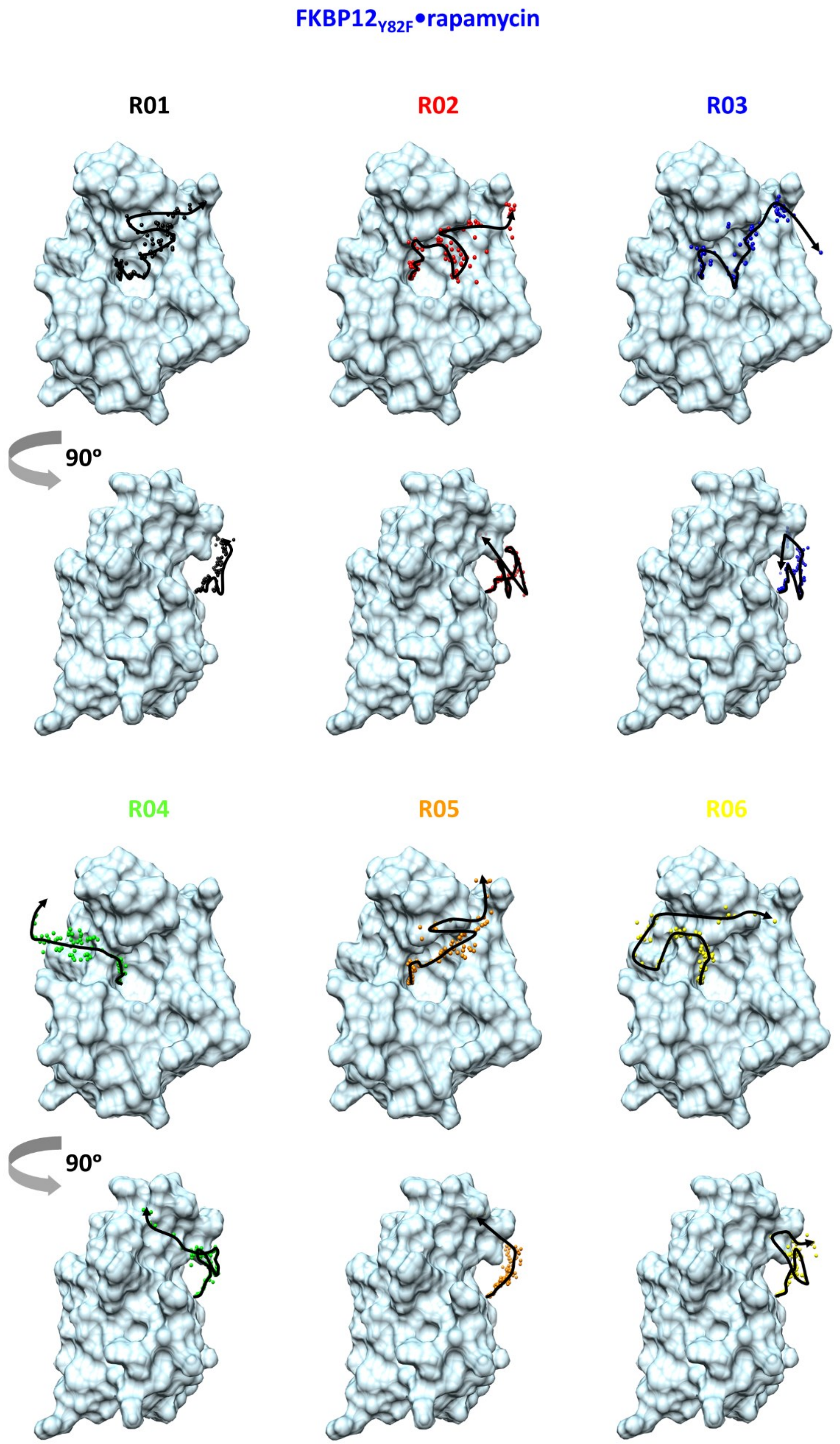
FKBP12Y82F•rapamycin
R01
R02
R03
90º
R04
R05
R06
90º

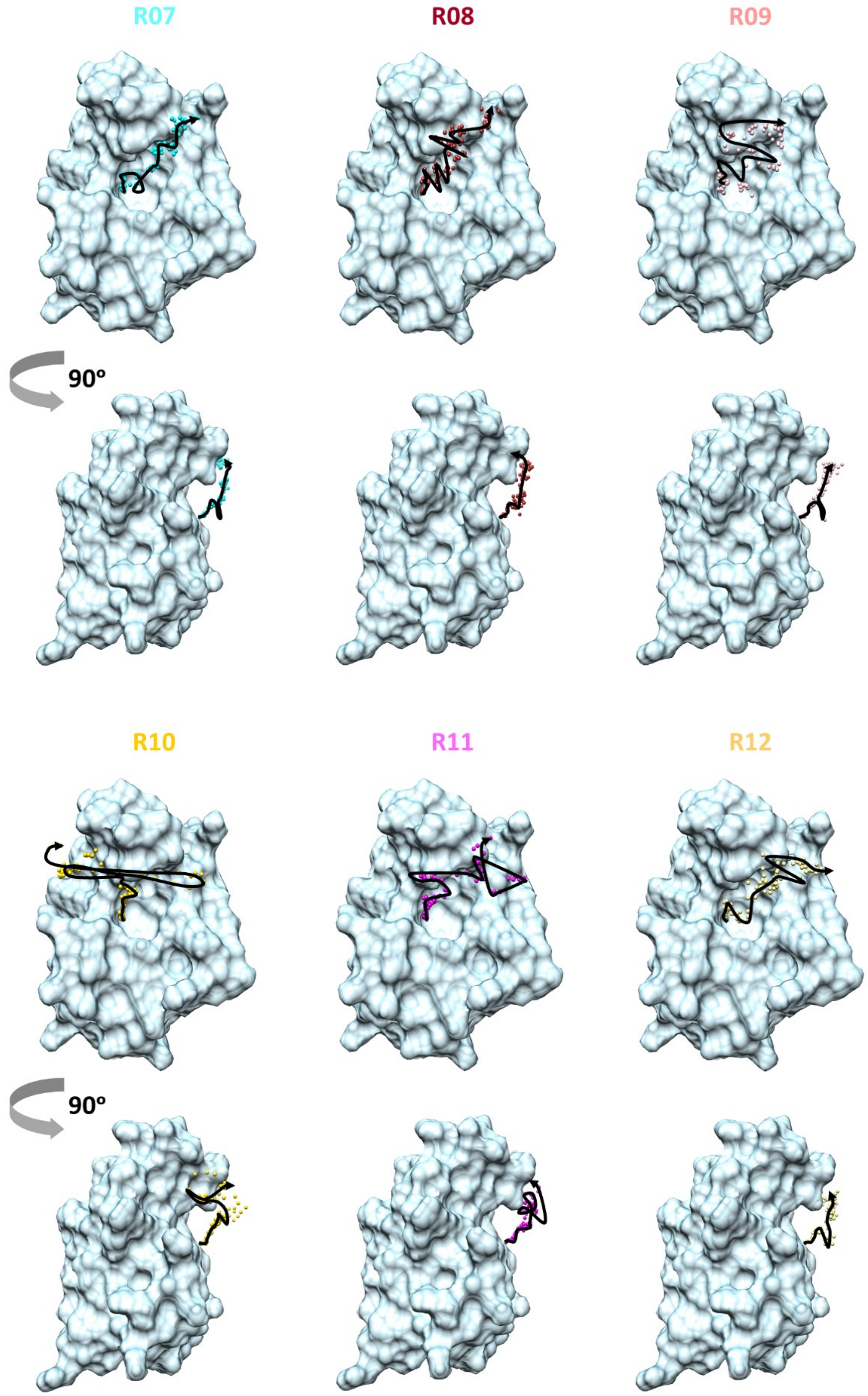
R07
R08
R09
90º
R10
R11
R12
90º

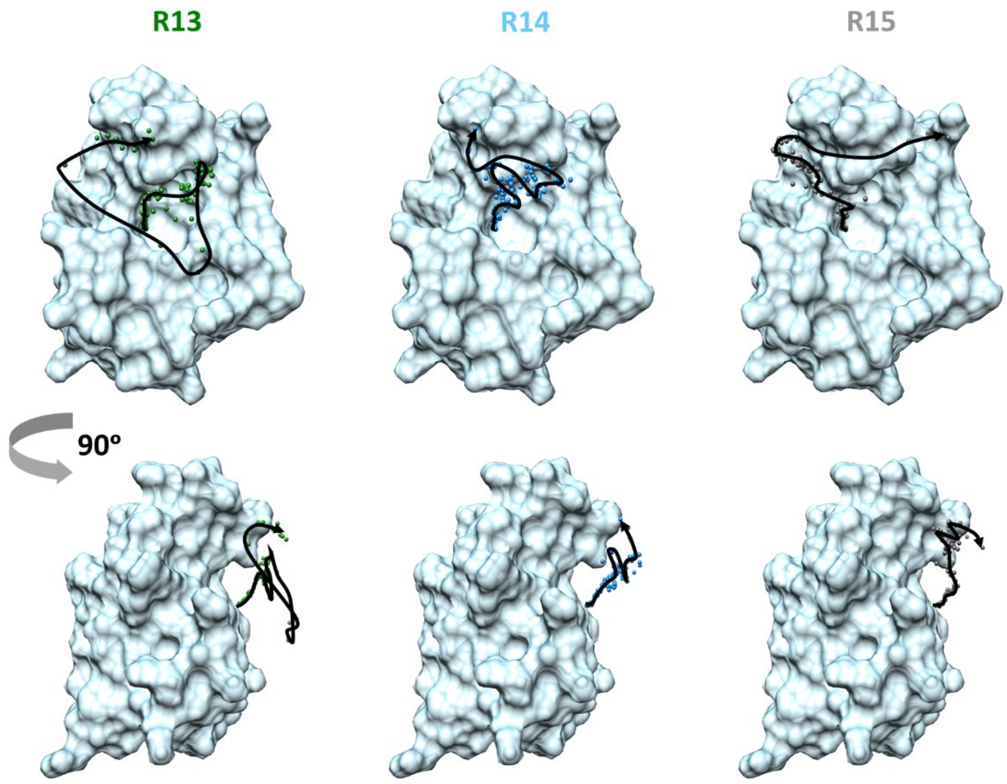

SUPPLEMENTARY FIGURE S12 | Curvilinear paths obtained for the $FKBP12_{Y82F}$•rapamycin → $FKBP12_{Y82F}$ + rapamycin dissociation. Each sphere roughly corresponds to the barycenter of all rapamycin CoG extracted from one umbrella sampling window of CPUS MD simulations. It has the same color than the PMF profile it is associated with. The black lines are guides for the eye. Data are only displayed up to $\boldsymbol{r}$ = 10 Å.

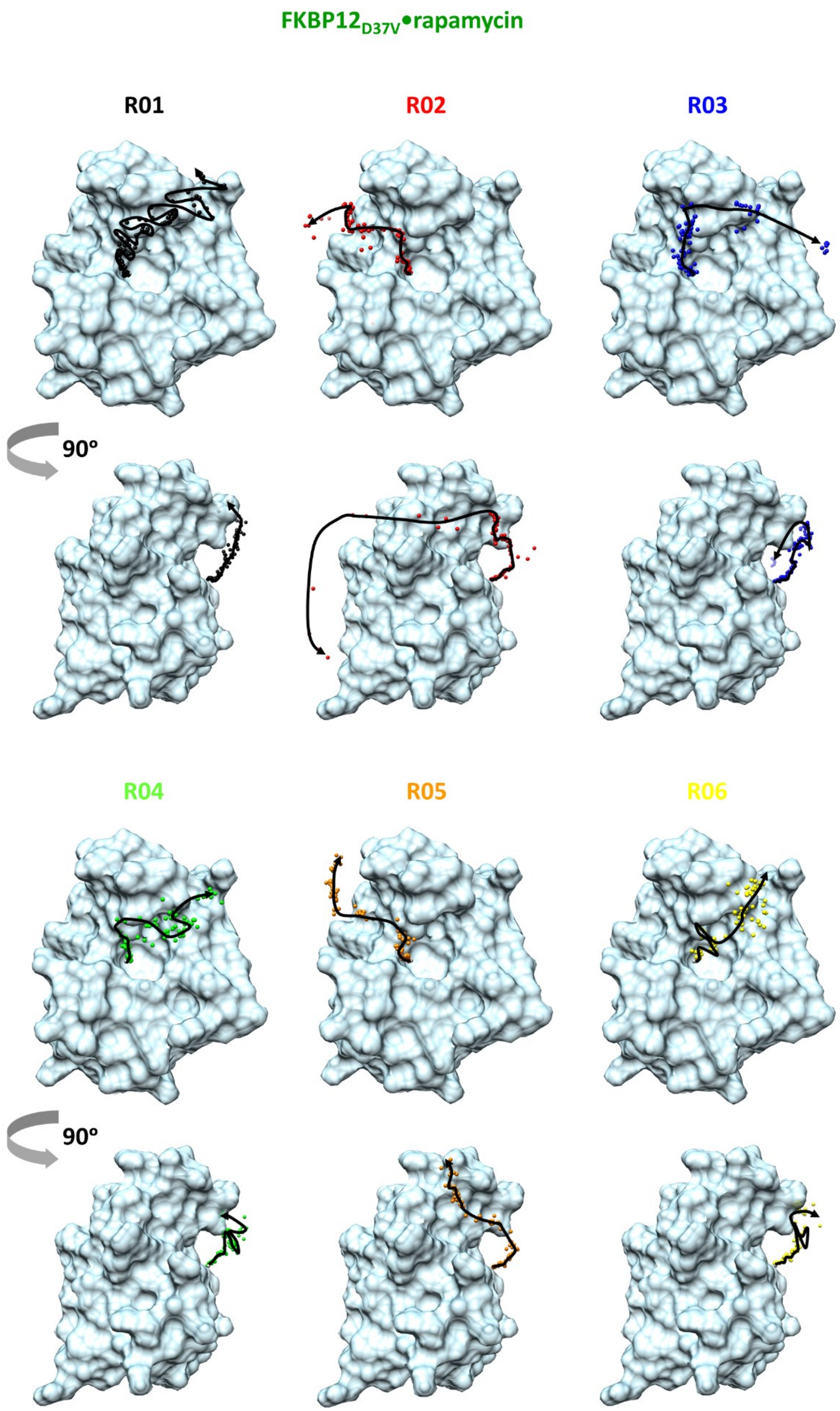
FKBP12$_{D37V}$•rapamycin
R01
R02
R03
90º
R04
R05
R06
90º

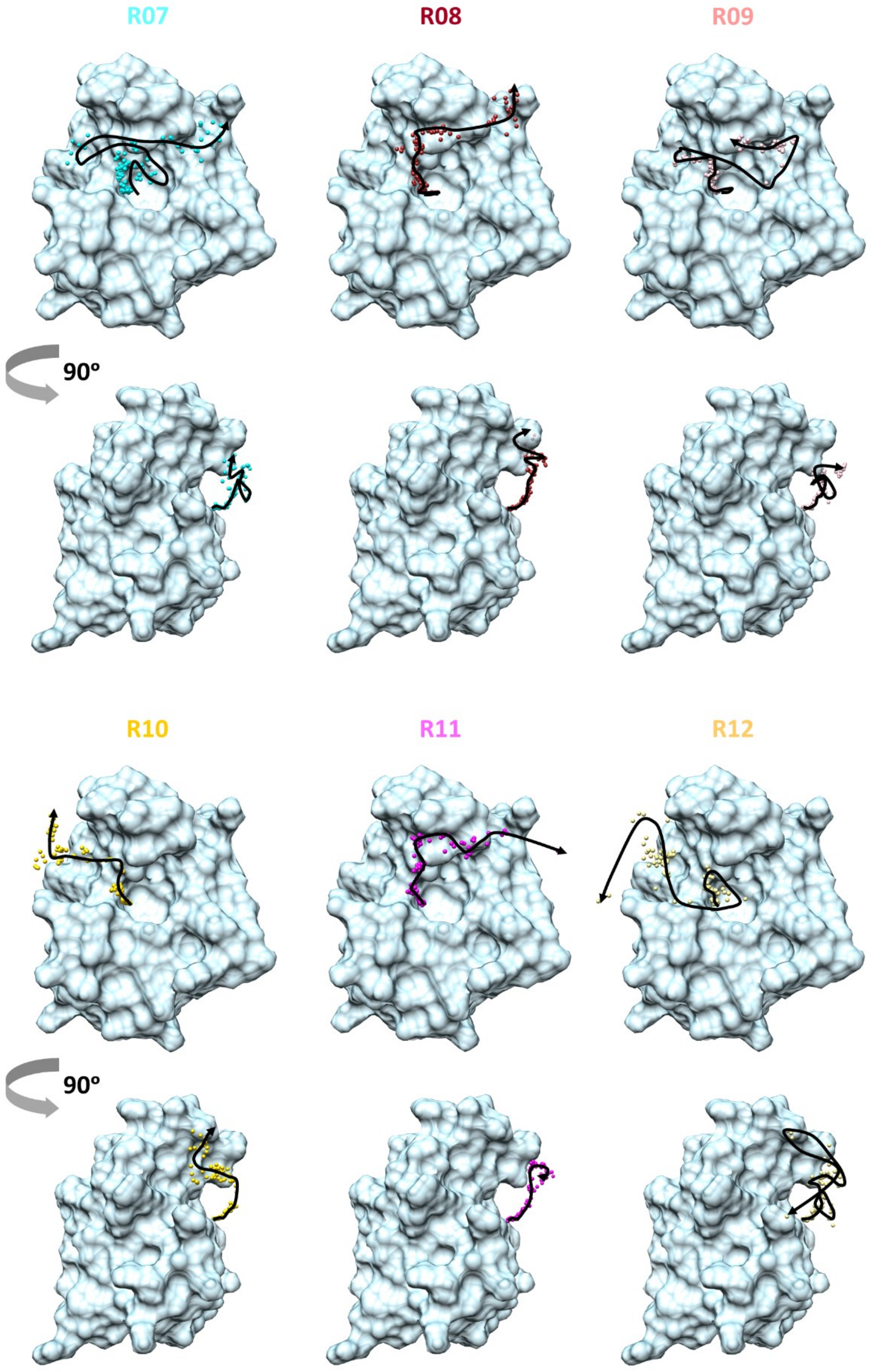
R07
R08
R09
90º
R10
R11
R12
90º

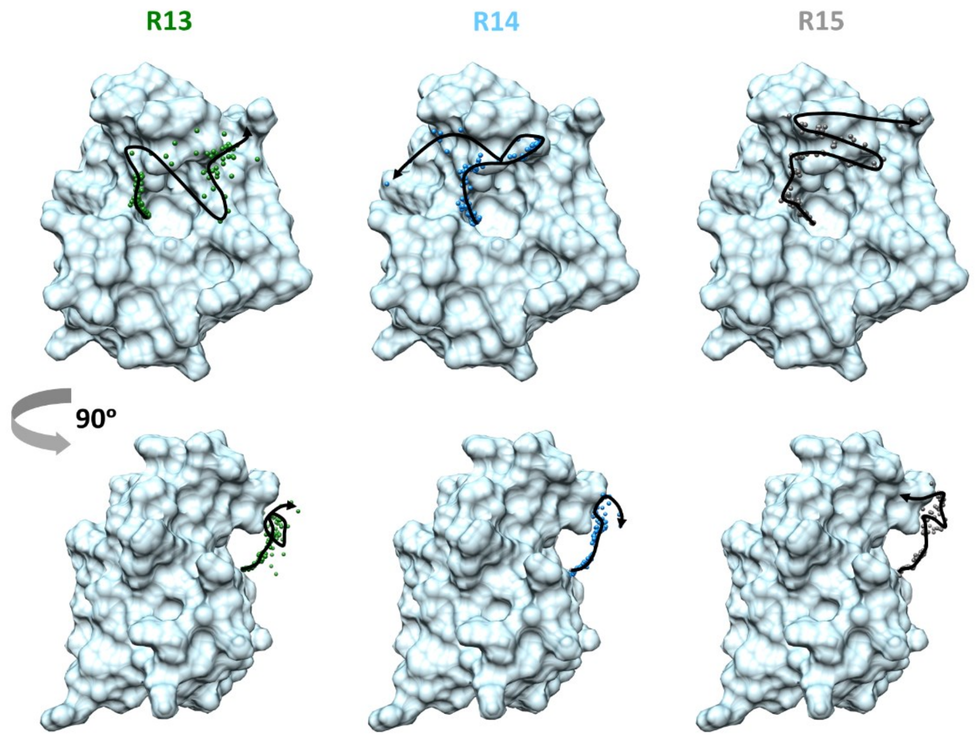

SUPPLEMENTARY FIGURE S13 | Curvilinear paths obtained for the FKBP12$_{D37V}$•rapamycin → FKBP12$_{D37V}$ + rapamycin dissociation. Each sphere roughly corresponds to the barycenter of all rapamycin CoG extracted from one umbrella sampling window of CPUS MD simulations. It has the same color than the PMF profile it is associated with. The black lines are guides for the eye. Data are only displayed up to $\boldsymbol{r}$ = 10 Å.

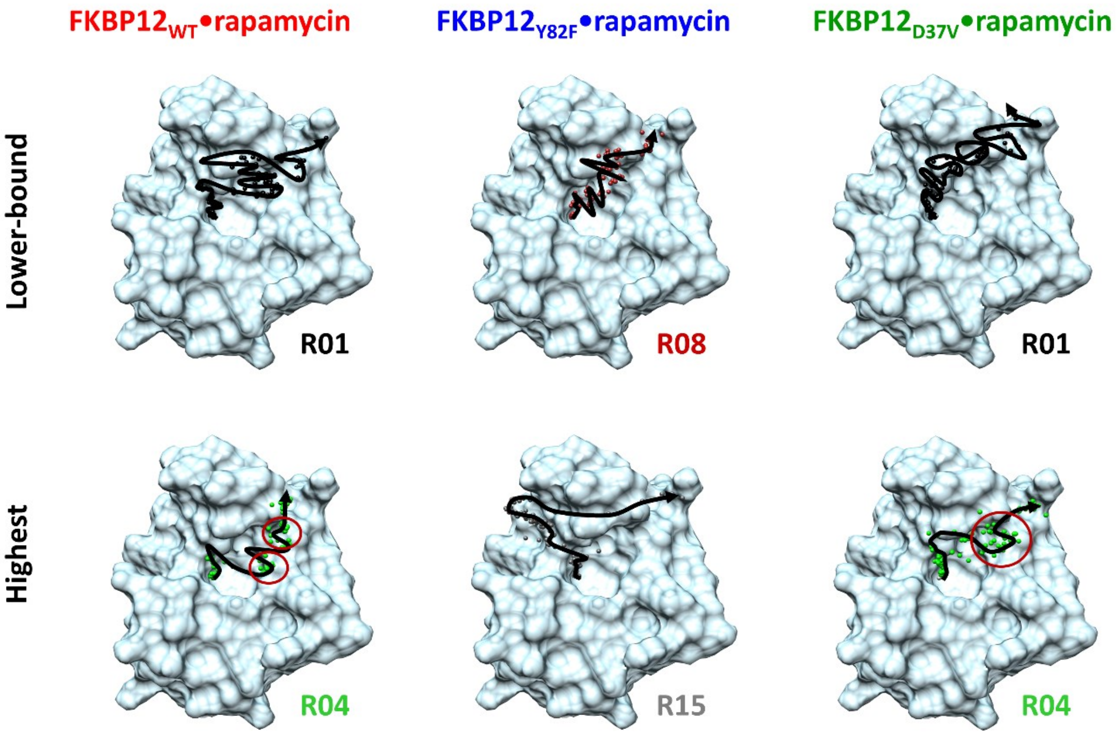

SUPPLEMENTARY FIGURE S14 | Comparison between the curvilinear paths of dissociation corresponding to lower-bound PMF profiles (top) and to highest PMF profiles (bottom). Images for $FKBP12_{WT}$•rapamycin (left), $FKBP12_{Y82F}$•rapamycin (middle), and $FKBP12_{D37V}$•rapamycin (right) were extracted from **Supplementary Figures S11**, **S12**, and **S13**, respectively. Red circles highlight stagnation areas along the dissociation paths.

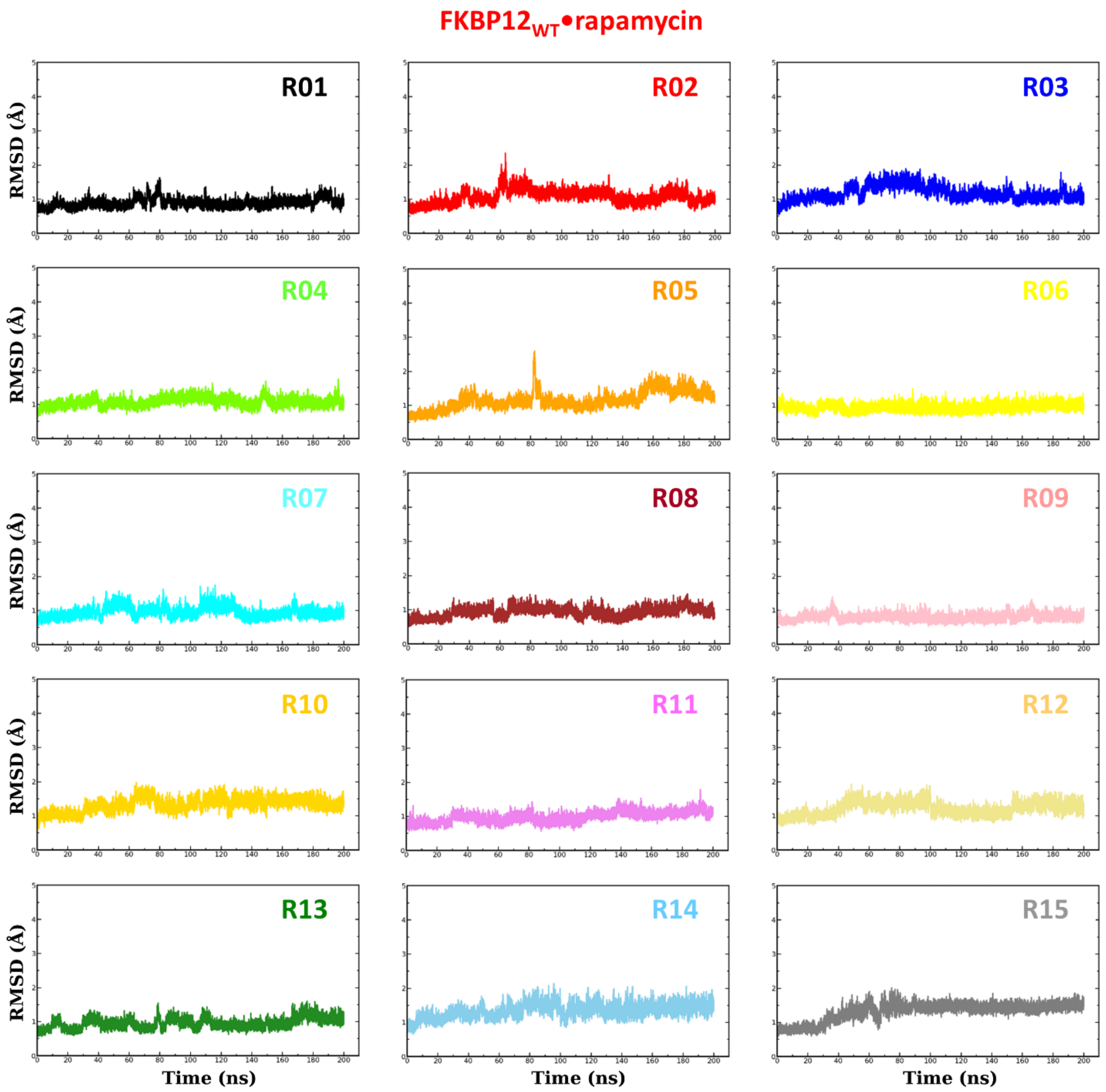

SUPPLEMENTARY FIGURE S15 | Evolution of rapamycin conformation during its dissociation from $FKBP12_{WT}$. The root-mean-square deviation was computed on all the heavy atoms of the macrolide (i.e. C, N, and O) and the conformation in the $FKBP12_{WT}$•rapamycin crystal pose (PDB 1FKB) was taken as reference. Pulling on the molecular partners CoG starts at 0 ns.

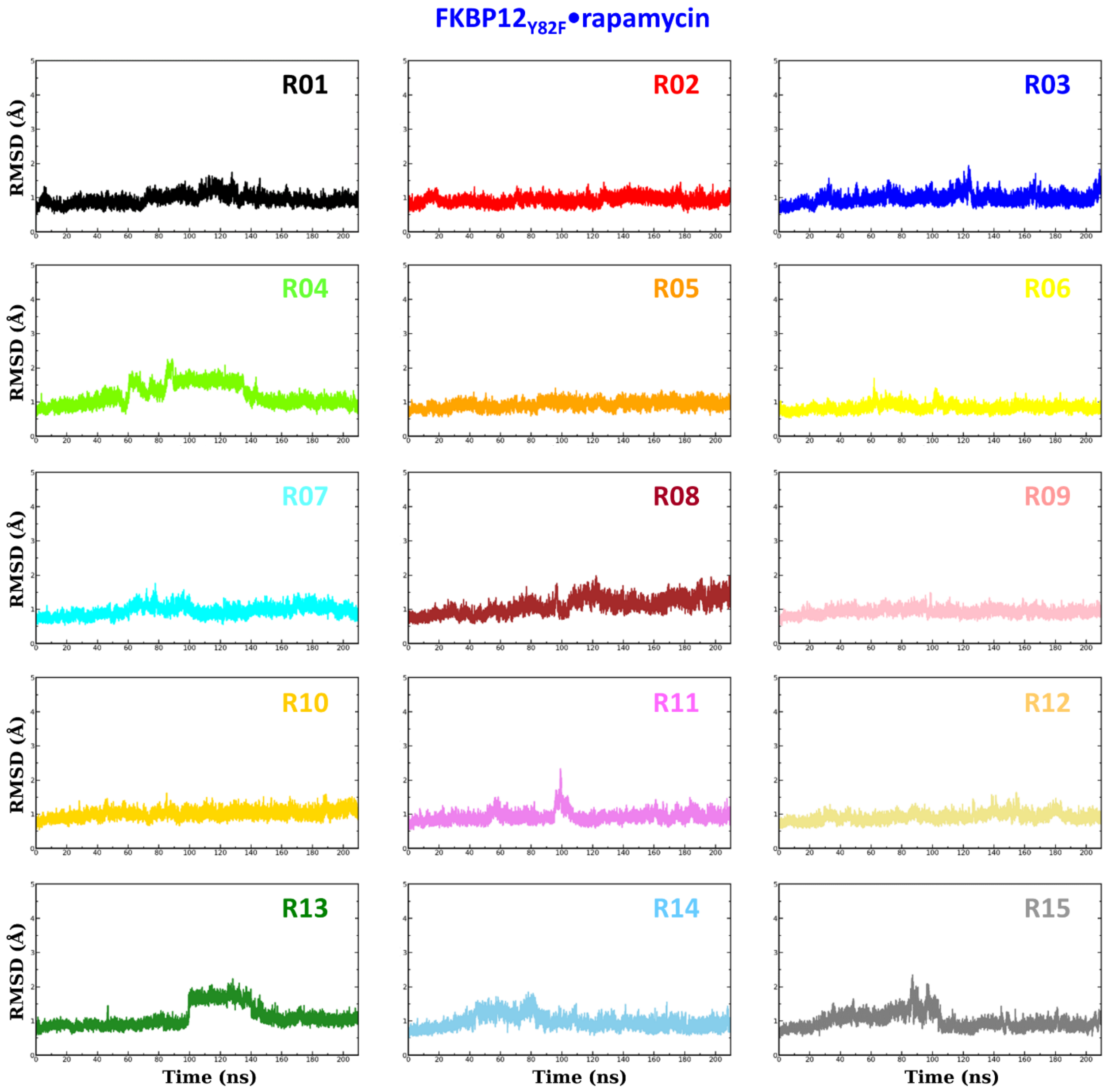

SUPPLEMENTARY FIGURE S16 | Evolution of rapamycin conformation during its dissociation from FKBP12$_{Y82F}$. The root-mean-square deviation was computed on all the heavy atoms of the macrolide (i.e. C, N, and O) and the conformation in the FKBP12$_{WT}$•rapamycin crystal pose (PDB 1FKB) was taken as reference. Pulling on the molecular partners CoG starts at 0 ns.

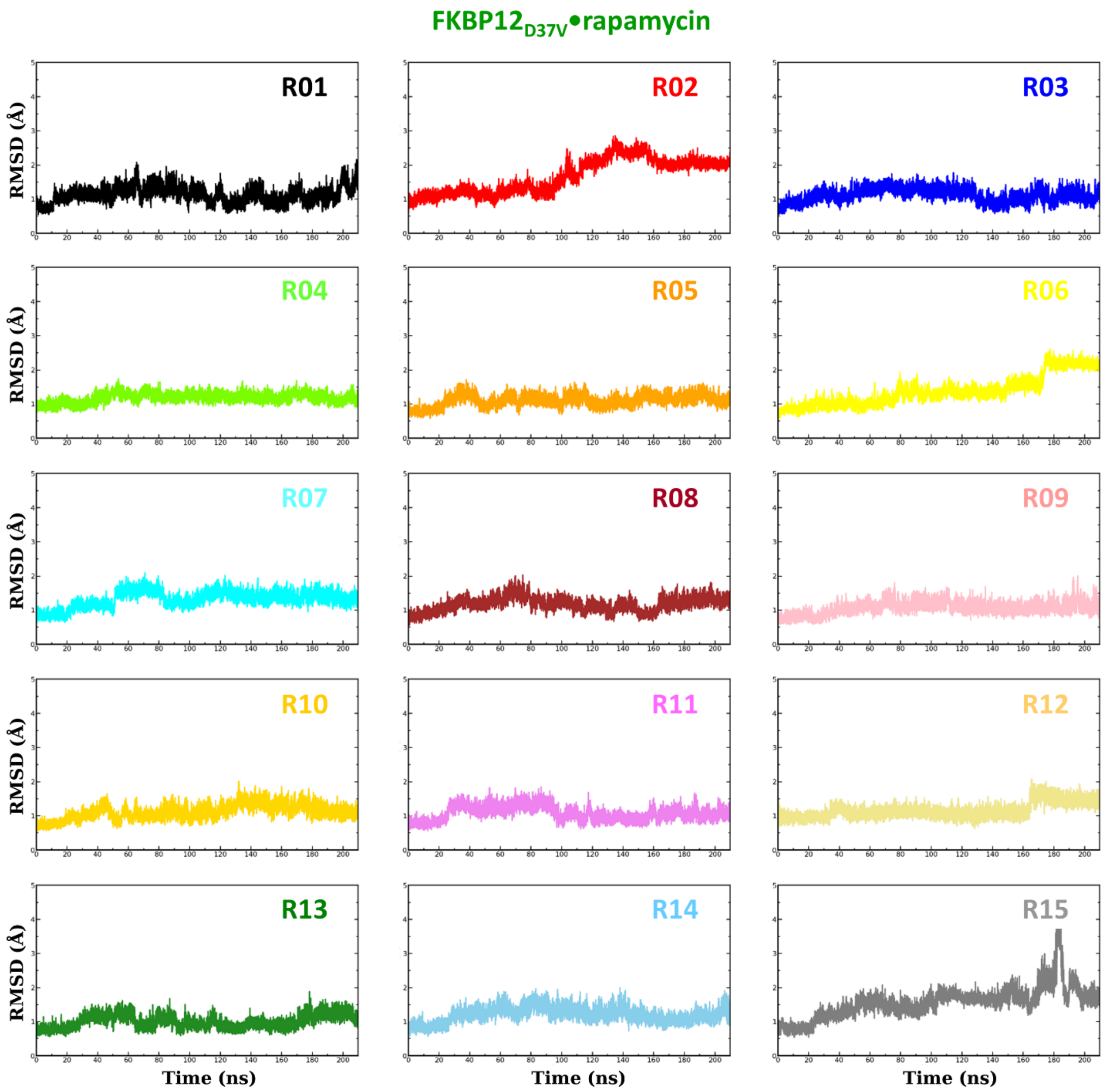

SUPPLEMENTARY FIGURE S17 | Evolution of rapamycin conformation during its dissociation from FKBP12$_{D37V}$. The root-mean-square deviation was computed on all the heavy atoms of the macrolide (i.e. C, N, and O) and the conformation in the FKBP12$_{WT}$•rapamycin crystal pose (PDB 1FKB) was taken as reference. Pulling on the molecular partners CoG starts at 0 ns.

SUPPLEMENTARY TABLE S1 | Cutoff separation distance and binding free energies obtained from the 15 independent runs of CPUS MD simulations performed on the complexes formed between rapamycin and each of the three FKBP12 variants. The lower-bound PMFs have been highlighted in bold and with the same color as in the plots (see **Figure 2**).

Run index	$FKBP12_{WT}$•rapamycin			$FKBP12_{Y82F}$•rapamycin			$FKBP12_{D37V}$•rapamycin		
	ΔG_{PMF} (kcal/mol)	$r_{*,cut}$ (Å)	ΔG^0_{bind} (kcal/mol)	ΔG_{PMF} (kcal/mol)	$r_{*,cut}$ (Å)	ΔG^0_{bind} (kcal/mol)	ΔG_{PMF} (kcal/mol)	$r_{*,cut}$ (Å)	ΔG^0_{bind} (kcal/mol)
R01	**-11.09**	**12.89**	**-13.98**	-11.69	14.59	-14.84	**-8.08**	**13.97**	**-10.78**
R02	-13.87	15.64	-17.24	-11.80	13.95	-14.85	-12.21	15.12	-15.39
R03	-12.68	16.58	-16.07	-12.43	18.44	-16.03	-12.95	12.69	-15.76
R04	-15.74	14.44	-18.98	-14.23	17.94	-17.85	-13.96	15.48	-17.27
R05	-13.31	18.86	-16.96	-10.43	17.60	-13.87	-9.25	16.96	-12.45
R06	-11.38	17.39	-14.84	-13.34	13.59	-16.42	-12.03	14.69	-15.19
R07	-12.63	18.79	-16.29	-12.69	17.90	-16.23	-10.03	12.17	-12.67
R08	-11.73	18.40	-15.35	**-10.21**	**14.38**	**-13.22**	-12.03	15.33	-15.22
R09	-13.8	15.69	-17.13	-12.12	16.99	-15.56	-10.14	16.59	-13.36
R10	-11.89	18.76	-15.51	-13.09	13.40	-16.11	-12.21	16.41	-15.55
R11	-12.16	14.93	-15.34	-12.45	18.36	-16.11	-12.05	17.95	-15.50
R12	-13.75	18.17	-17.39	-11.23	17.00	-14.64	-11.68	15.43	-14.87
R13	-12.11	14.10	-15.18	-13.83	18.57	-17.52	-10.33	14.02	-13.24
R14	-12.55	16.77	-16.00	-10.94	15.51	-14.09	-11.41	16.50	-14.63
R15	-14.88	15.95	-18.30	-17.60	17.86	-21.32	-10.66	16.33	-13.90

SUPPLEMENTARY TABLE S2 | Published thermodynamic parameters for the three variants of FKBP12•rapamycin ⇄ FKBP12 + rapamycin reaction. K_{exp}^{meas} corresponds to the dissociation equilibrium constant measured at temperature T^{meas} , $-\Delta G_{exp}^{meas}$ to the dissociation free energy at the same temperature, and $-\Delta G_{exp}^{corr}$ to the latter parameter corrected so as to be compared with the MD CPUS simulations performed at temperature T^{sim} = 21.85 °C. [a,b,c]

FKBP12$_{WT}$•rapamycin ⇄ FKBP12$_{WT}$ + rapamycin

Assay [d]	Monitoring technique	Buffer [e]	T^{meas} (°C) [f]	K_{exp}^{meas} (nM) [g]	$-\Delta G_{exp}^{meas}$ (kcal/mol)	$-\Delta G_{exp}^{corr}$ (kcal/mol)	Reference	Data index [h]
Competitive titration using FKBP12•[^{3}H]dihydroFK506	Gel filtration and scintillation spectrometry	20 mM PB pH 7.3<br>100 mM NaCl<br>1 mM EDTA	NS	0.2	13.11	13.11	Bierer et al. 1990 [6]	1
Inhibition of the rotamase activity (Suc-AlaLeuProPhe-pNA substrate)	Product hydrolysis by α-chimotrypsin and UV-vis absorption spectrometry	40 mM HEPES pH 7.9	10	0.2	12.58	12.36	Bierer et al. 1990 [6]	1
Inhibition of the rotamase activity (Suc-AlaPheProPhe-pNA substrate)	Product hydrolysis by α-chimotrypsin and UV-vis absorption spectrometry	35 mM HEPES pH 7.8	10	0.3 – 0.6	12.13 ± 0.19	11.89 ± 0.20	Holt et al. 1993 [7]	2
Inhibition of the rotamase activity (Suc-AlaLeuProPhe-pNA and Suc-PheLeuProPhe-pNA substrates)	Product hydrolysis by α-chimotrypsin and UV-vis absorption spectrometry	35 mM HEPES pH 7.8	10	0.3 – 0.6<br>0.4 ± 0.2	12.13 ± 0.19<br>12.19 ± 0.28	11.89 ± 0.20<br>11.95 ± 0.29	Bossard et al. 1994 [8]	2
Inhibition of the rotamase activity (Suc-AlaPheProPhe-pNA substrate)	Product hydrolysis by α-chimotrypsin and UV-vis absorption spectrometry	35 mM HEPES pH 7.8	10	0.6	11.96	11.72	Luengo et al. 1995 [9]	2
Inhibition of the rotamase activity (Suc-AlaLeuProPhe-pNA substrate)	Product hydrolysis by α-chimotrypsin and UV-vis absorption spectrometry	100 mM Tris pH 7.8	15	0.26 ± 0.10	12.66 ± 0.22	12.53 ± 0.23	DeCenzo et al. 1996 [10]	3
Competitive titration using immobilized BSA-FK506•FKBP12-Biotin	ELISA relying on Streptavidin-AP and UV-vis absorption spectrometry	NS	NS	0.4 – 0.9	12.42 ± 0.23	12.42 ± 0.23	Schuler et al. 1997 [11]	4
Competitive titration using immobilized FKBP12-CSK•ascomycin-AP	UV-vis absorption spectrometry	PBS pH 7.4	RT	1.1 ± 0.5	12.11 ± 0.27	12.11 ± 0.27	Wagner et al. 1998 [12]	5

Competitive titration using immobilized His_6-FKBP12•[^{3}H]dihydroFK506	Proximity scintillation assay on anti-His_6 coated beads	50 mM HEPES pH 7.4	RT	3.5	11.43	11.43	Graziani et al. 1999 [13]	6
Competitive titration using immobilized FKBP12-CSK•ascomycin-AP	UV-vis absorption spectrometry	PBS pH 7.4	RT	1.6	11.89	11.89	Dickman et al. 2000 [14]	5
Competitive titration using FKBP12•Fluorescein-SLF	Fluorescence polarization [K for FKBP12•Fluorescein-SLF from Ref. 15]	PBS pH 7.4	NS	[0.35]	12.78	12.78	Banaszynski et al. 2005 [16]	7
Titration of immobilized GST-FKBP12	Surface plasmon resonance on carboxymethylated dextran surfaces	PBS pH 7.4	25	0.27	13.07	13.12	Banaszynski et al. 2005 [16]	7
Inhibition of the rotamase activity for both FKBP12 and His_6-FKBP12 (Suc-AlaLeuProPhe-pNA substrate)	Product hydrolysis by α-chimotrypsin and UV-vis absorption spectrometry	50 mM HEPES pH 8.0<br>100 mM NaCl	4	7.5 ± 1.9<br>6.1 ± 2.1	10.32 ± 0.14<br>10.43 ± 0.19	9.83 ± 0.15<br>9.95 ± 0.20	Wear et al. 2007 [17]	8
Titration of FKBP12 and His_6-FKBP12	Intrinsic fluorescence intensity of Trp59	50 mM HEPES pH 8.0<br>100 mM NaCl<br>0.5 mM EDTA	25	8.7 ± 3.5<br>6.8 ± 1.2	11.01 ± 0.24<br>11.16 ± 0.10	11.08 ± 0.24<br>11.23 ± 0.10	Wear et al. 2007 [17]	8
Titration by FKBP12 and His_6-FKBP12 of rapamycin	Isothermal titration calorimetry	50 mM HEPES pH 8.0<br>100 mM NaCl<br>0.5 mM EDTA	NS	2.0 ± 0.5<br>3.7 ± 1.2	11.76 ± 0.15<br>11.40 ± 0.19	11.76 ± 0.15<br>11.40 ± 0.19	Wear et al. 2007 [17]	8
Titration of immobilized His_6-FKBP12	Surface plasmon resonance on Streptavidin•Biotin-NTA-Ni^{2+} and NTA-Ni^{2+} surfaces	10 mM HEPES pH 7.4<br>150 mM NaCl<br>0.05 mM EDTA	NS	2.13 ± 0.36<br>1.67 ± 0.1	11.72 ± 0.10<br>11.86 ± 0.04	11.72 ± 0.10<br>11.86 ± 0.04	Wear & Walkinshaw 2007 [18]	8
Titration of His_6-FKBP12	Isothermal titration calorimetry	10 mM HEPES pH 7.4<br>150 mM NaCl<br>0.05 mM EDTA	25	2.74 ± 0.54	11.70 ± 0.12	11.76 ± 0.12	Wear & Walkinshaw 2007 [18]	8
Competitive titration using His_6-FKBP12•[^{3}H]dihydroFK506	Proximity scintillation assay on Ni-chelate surfaces	50 mM HEPES pH 7.5	NS	((2))	11.76	11.76	Shor et al. 2008 [19]	9
Competitive titration using FKBP12•Fluorescein-rapamycin	Fluorescence polarization	20 mM HEPES pH 8.0	RT	0.60 ± 0.17	12.47 ± 0.17	12.47 ± 0.17	Kozany et al. 2009 [20]	10
Competitive titration using FKBP12•Fluorescein-SLF	Fluorescence polarization [K for FKBP12•Fluorescein-SLF from Ref. 15]	12 mM PB pH 7.4<br>137 mM NaCl<br>2.7 mM KCl	RT	[57 ± 20]	9.79 ± 0.21	9.79 ± 0.21	Wu et al. 2011 [21]	11
Competitive titration using OregonGreen-FKBP12•Ts-SLF	Fluorescence intensity [K for FKBP12•SLF from Ref. 22]	50 mM HEPES pH 7.2	25	[0.3]	13.01	13.06	Tamura et al. 2013 [23]	12

Titration by FKBP12 of immobilized rapamycin	Surface plasmon resonance imaging on 3D and 2D photo-cross-linked slides	PBS pH 7.4	25	1.75<br>17.5	11.96<br>10.60	12.03<br>10.67	Singh et al. 2015 [24]	13
Titration of a FKBP12-CyPet and FRB- YPet mixture	Fluorescence intensity (low reliability fit)	PBS pH 7.4	25	(2.1)	11.86	11.92	Lu & Wang 2017 [25]	14
Lifetime evaluation with tethered FKBP12 and FRB manipulated thanks to DNA handles	Single-molecule assay in force cycling mode and at 1 nM rapamycin	PBS	NS	0.6 ± 0.1	12.47 ± 0.10	12.46 ± 0.10	Wang et al. 2019 [26]	15
Titration of FKBP12 and FRB engrafted on a DNA scaffold	Single-molecule assay in fluctuation mode and at 0.05 to 0.2 nM rapamycin	20 mM HEPES pH 7.8<br>100 mM KCl<br>5 mM $MgCl_2$	21.7	0.28 ± 0.04	12.91 ± 0.08	12.90 ± 0.08	Kostrz et al. 2019 [3]	16

$FKBP12_{Y82F}$•rapamycin ⇄ $FKBP12_{Y82F}$ + rapamycin

Assay	Monitoring technique	Buffer	T^{meas} (°C)	K_{exp}^{meas} (nM)	$-\Delta G_{exp}^{meas}$ (kcal/mol)	$-\Delta G_{exp}^{corr}$ (kcal/mol)	Reference	Data index
Titration by FKBP12 of rapamycin	Isothermal titration calorimetry	50 mM PB pH 7<br>50 mM NaCl	25	4.4	11.42	11.51	Connelly et al. 1994 [27]	17
Inhibition of the rotamase activity (Suc-AlaLeuProPhe-pNA substrate)	Product hydrolysis by α-chimotrypsin and UV-vis absorption spectrometry	100 mM Tris pH 7.8	15	0.60 ± 0.24	12.18 ± 0.23	11.98 ± 0.23	DeCenzo et al. 1996 [10]	3

$FKBP12_{D37V}$•rapamycin ⇄ $FKBP12_{D37V}$ + rapamycin

Assay	Monitoring technique	Buffer	T^{meas} (°C)	K_{exp}^{meas} (nM)	$-\Delta G_{exp}^{meas}$ (kcal/mol)	$-\Delta G_{exp}^{corr}$ (kcal/mol)	Reference	Data index
Inhibition of the rotamase activity (Suc-AlaLeuProPhe-pNA substrate)	Product hydrolysis by α-chimotrypsin and UV-vis absorption spectrometry	100 mM Tris pH 7.8	15	36.0 ± 5.8	9.83 ± 0.09	9.61 ± 0.09	DeCenzo et al. 1996 [10]	3
Titration by FKBP12 of immobilized rapamycin	Surface plasmon resonance imaging on 3D photo-cross-linked slides	PBS pH 7.4	25	55.3	9.91	10.01	Singh et al. 2015 [24]	13

[a] Abbreviations: FK506 = fujimycin = tacrolimus; Suc = succinyl, pNA = *para*-nitroanilide; BSA = bovine serum albumin; AP = alkaline phosphatase; CSK = CMP-KDO synthetase; SLF = synthetic ligand for FKBP12; GST = glutathione S-transferase; Ts = tosylic acid; CyPet = cyan fluorescent protein for energy transfer; YPet = yellow fluorescent protein for energy transfer; ELISA = enzyme-linked immunosorbent assay; PB = phosphate buffer; PBS = phosphate buffer saline.

[b] Binding free energies were computed as $\Delta G_{exp}^{meas} = RT^{meas}$ ln$[K_{exp}^{meas}]$ and $\Delta G_{exp}^{corr} = RT^{sim}$ ln$[K_{exp}^{meas}] + \Delta H_{exp} \times (1 - T^{sim}/T^{meas})$ with R = 1.99 cal mol^{-1} K^{-1}, T^{sim} = 21.85 °C = 295 K, as well as ΔH_{exp} = -17.90 ± 0.38 kcal/mol and -20.33 ± 0.34 kcal/mol for the wild-type protein and for the Y82F mutant, respectively (measurements performed at 20.5 °C).[27, 28] The enthalpy of binding is not available for the D37V mutant, we thus used the -19 ± 1 kcal/mol intermediate value. No error bar is usually provided for T^{meas} but, as can be seen on **Supplementary Figure S8**, the usual ± 0.1 to ± 0.5 °C specification given for most apparatuses would result in minute changes for the binding free energy. When the error on K_{exp}^{meas} was specified it was propagated according to $\sigma_{\Delta G_{exp}^{meas}} = RT^{meas}\, \sigma_{K_{exp}^{meas}}/K_{exp}^{meas}$ and $\sigma_{\Delta G_{exp}^{corr}}{}^2 = (RT^{sim}\, \sigma_{K_{exp}^{meas}}/K_{exp}^{meas})^2 + \sigma_{\Delta H_{exp}}{}^2 \times (1 - T^{sim}/T^{meas})^2$.[29] Conversely, we did not report any error on ΔG_{exp}^{corr} when the uncertainty on $\sigma_{K_{exp}^{meas}}$ was not indicated; indeed, the influence of $\sigma_{\Delta H_{exp}}$ is very limited and the resulting small $\sigma_{\Delta G_{exp}^{corr}}$ values would not account for the reality of the measurements. When a range was provided for K_{exp}^{meas} we first converted it by taking the mean of the extremal values as a central value and the full range as error bar, then we performed the computation of both ΔG_{exp}^{meas} and ΔG_{exp}^{corr}.

[c] Rapamycin = sirolimus is registered under ID BDBM36609 in BindingDB (www.bindingdb.org) and all values reported in this databank have been included in the present Table.

[d] Unless otherwise specified, the titrant is rapamycin. In inhibition assays it competes with peptidic substrates of FKBP12 and thus prevents their isomerization; in competitive titrations it binds to the same pocket as the tracers and thus favors the disruption of the indicated complexes. Various mathematical models can be used to analyze the results of these assays and sometimes the authors do not provide the equilibrium dissociation constant but parameters such as the inhibitor constant, K_i, or the half maximal inhibitory concentration activity, IC_{50}. Although the two latter parameters are not identical to K it is here assumed that if appropriate conditions have been selected to run the experiment they offer a fair estimate of it (at least within the same order of magnitude).

[e] Only the buffering system and the major salts are provided. Among the other components one may find DTT and EDTA (at millimolar concentrations), glycerol used for stabilization purpose (at up to more than 10 %), and organic solvents (e.g. methanol, ethanol, or DMSO) used to dissolve rapamycin (at up to a few %).

[f] This parameter is sometimes not specified (NS) or just indicated as "room temperature" (RT); in both cases, we assumed measurements were performed around 20 – 25 °C, i.e. $T^{meas} = T^{sim}$ = 21.85 °C. As evidenced on **Supplementary Figure S8** a 2 to 3 °C imprecision on temperature should not result in too large changes for the binding free energy.

[g] Values between parentheses are hypothesized to be of low quality by the researchers that collected them, the reason of which been given in the adjacent column. Values between double parentheses were inferred by ourselves from the graphical data displayed in the cited article. Values between brackets partly rely on data sets published in other articles, the corresponding reference been given in the adjacent column.

[h] This index is used to plot **Figure 2D** and **Supplementary Figure S8**.

SUPPLEMENTARY BIBLIOGRAPHY

1. Wallace, A. C., Laskowski, R. A. & Thornton, J. M. LIGPLOT - A program to generate schematic diagrams of protein ligand interactions. *Protein Eng.* **8**, 127-134 (1995).
2. Van Duyne, G. D., Standaert, R. F., Schreiber, S. L. & Clardy, J. Atomic-structure of the rapamycin human immunophilin FKBP-12 complex. *J. Am. Chem. Soc.* **113**, 7433-7434 (1991).
3. Kostrz, D. et al. A modular DNA scaffold to study protein-protein interactions at single-molecule resolution. *Nat. Nanotechnol.* **14**, 988-993 (2019).
4. Williamson, M. P. Chemical shift perturbation *in* Modern magnetic resonance. (ed. G.A. Webb) 995-1012 (Springer International Publishing, Cham; 2018).
5. Ayed, A. et al. Latent and active p53 are identical in conformation. *Nat. Struct. Biol.* **8**, 756-760 (2001).
6. Bierer, B. E. et al. Two distinct signal transmission pathways in T lymphocytes are inhibited by complexes formed between an immunophilin and either FK506 or rapamycin. *Proc. Natl. Acad. Sci. U. S. A.* **87**, 9231-9235 (1990).
7. Holt, D. A. et al. Design, synthesis, and kinetic evaluation of high-affinity fkbp ligands and the x-ray crystal-structures of their complexes with FKBP12. *J. Am. Chem. Soc.* **115**, 9925-9938 (1993).
8. Bossard, M. J. et al. Catalytic and ligand-binding properties of the FK506 binding-protein FKBP12 - Effects of the single amino-acid substitution of Tyr(82) to Leu. *Biochem. J.* **297**, 365-372 (1994).
9. Luengo, J. I. et al. structure-activity studies of rapamycin analogs - Evidence that the C-7 methoxy group is part of the effector domain and positioned at the FKBP12-FRAP interface. *Chem. Biol.* **2**, 471-481 (1995).
10. DeCenzo, M. T. et al. FK506-binding protein mutational analysis: Defining the active-site residue contributions to catalysis and the stability of ligand complexes. *Protein Eng.* **9**, 173-180 (1996).
11. Schuler, W. et al. SDZ RAD, a new rapamycin derivative - Pharmacological properties in vitro and in vivo. *Transplantation* **64**, 36-42 (1997).
12. Wagner, R. et al. 32-ascomycinyloxyacetic acid derived immunosuppressants. Independence of immunophilin binding and immunosuppressive potency. *J. Med. Chem.* **41**, 1764-1776 (1998).
13. Graziani, F., Aldegheri, L. & Terstappen, G. C. High throughput scintillation proximity assay for the identification of FKBP-12 ligands. *J. Biomol. Screen* **4**, 3-7 (1999).
14. Dickman, D. A. et al. Antifungal rapamycin analogues with reduced immunosuppressive activity. *Bioorg. Med. Chem. Lett.* **10**, 1405-1408 (2000).
15. Braun, P. D. & Wandless, T. J. Quantitative analyses of bifunctional molecules. *Biochemistry* **43**, 5406-5413 (2004).
16. Banaszynski, L. A., Liu, C. W. & Wandless, T. J. Characterization of the FKBP - Rapamycin - FRB ternary complex. *J. Am. Chem. Soc.* **127**, 4715-4721 (2005).
17. Wear, M. A., Patterson, A. & Walkinshaw, M. D. A kinetically trapped intermediate of FK506 binding protein forms in vitro: Chaperone machinery dominates protein folding in vivo. *Protein Expr. Purif.* **51**, 80-95 (2007).
18. Wear, M. A. & Walkinshaw, M. D. Determination of the rate constants for the FK506 binding protein/rapamycin interaction using surface plasmon resonance: An alternative sensor surface for Ni^{2+}-nitrilotriacetic acid immobilization of His-tagged proteins. *Anal. Biochem.* **371**, 250-252 (2007).
19. Shor, B. et al. A new pharmacologic action of CCI-779 involves FKBP12-independent inhibition of mTOR kinase activity and profound repression of global protein synthesis. *Cancer Res.* **68**, 2934-2943 (2008).
20. Kozany, C., Marz, A., Kress, C. & Hausch, F. Fluorescent probes to characterise FK506-binding proteins. *ChemBioChem* **10**, 1402-1410 (2009).

21. Wu, X. et al. Creating diverse target-binding surfaces on FKBP12: Synthesis and evaluation of a rapamycin analogue library. *ACS Comb. Sci.* **13**, 486-495 (2011).
22. Briesewitz, R., Ray, G. T., Wandless, T. J. & Crabtree, G. R. Affinity modulation of small-molecule ligands by borrowing endogenous protein surfaces. *Proc. Natl. Acad. Sci. U. S. A.* **96**, 1953-1958 (1999).
23. Tamura, T., Kioi, Y., Miki, T., Tsukiji, S. & Hamachi, I. Fluorophore labeling of native FKBP12 by ligand-directed tosyl chemistry allows detection of its molecular interactions in vitro and in living cells. *J. Am. Chem. Soc.* **135**, 6782-6785 (2013).
24. Singh, V., Nand, A. & Sarita Universal screening platform using three-dimensional small molecule microarray based on surface plasmon resonance imaging. *RSC Adv.* **5**, 87259-87265 (2015).
25. Lu, C. & Wang, Z. X. Quantitative analysis of ligand induced heterodimerization of two distinct receptors. *Anal. Chem.* **89**, 6926-6930 (2017).
26. Wang, Y. N. et al. Label-free single-molecule quantification of rapamycin-induced FKBP-FRB dimerization for direct control of cellular mechanotransduction. *Nano Lett.* **19**, 7514-7525 (2019).
27. Connelly, P. R. et al. Enthalpy of hydrogen-bond formation in a protein-ligand binding reaction. *Proc. Natl. Acad. Sci. U. S. A.* **91**, 1964-1968 (1994).
28. Connelly, P. R., Thomson, J. A., Fitzgibbon, M. J. & Bruzzese, F. J. Probing hydration contributions to the thermodynamics of ligand-binding by proteins - enthalpy and heat-capacity changes of tacrolimus and rapamycin binding to FK506 binding-protein in D_2O and H_2O. *Biochemistry* **32**, 5583-5590 (1993).
29. Bevington, P. R. & Robinson, D. K. Data reduction and error analysis for the physical sciences, Edn. 3rd. (McGraw-Hill, Boston; 2002).
